# Preliminary Quality and Safety Evaluation of Lycopene-Based Dietary Supplements: Analysis of Active Compound Content, Microbiological Purity, and Chemical Contaminants

**DOI:** 10.3390/foods15091583

**Published:** 2026-05-04

**Authors:** Kalina Sikorska-Zimny, Artur Miszczak, Wioletta Popińska, Paweł Lisiecki, Magdalena Szemraj, Oliwia Wojtasik, Patrycja Chmielewska, Katarzyna Wrzodak, Karolina Duda, Krzysztof P. Rutkowski, Małgorzata Wojciechowska

**Affiliations:** 1 Institute of Health Sciences, Medical, Natural and Technical College, Stefan Batory State University, 96-100 Skierniewice, Poland; pawel.lisiecki@umed.lodz.pl (P.L.); oliwia_wojtasik2005@wp.pl (O.W.); patchmielewska04@gmail.com (P.C.); kasia.wrzodak98@gmail.com (K.W.); karolinaduda181@gmail.com (K.D.); malgorzataw62@gmail.com (M.W.); 2 The National Institute of Horticultural Research, 96-100 Skierniewice, Poland; artur.miszczak@inhort.pl (A.M.); wioletta.popinska@inhort.pl (W.P.); krzysztof.rutkowski@inhort.pl (K.P.R.); 3 Department of Pharmaceutical Microbiology and Microbiological Diagnostics, Medical University of Łódź, 92-213 Łódź, Poland; magdalena.szemraj@umed.lodz.pl

**Keywords:** lycopene content in supplements, pesticide residues, heavy metal content, microbiology

## Abstract

Dietary supplements, especially lycopene-containing ones, are of interest because of their antioxidant and potential health-promoting effects; however, their actual composition and safety have not been sufficiently verified. This study evaluated the accuracy of labelled lycopene content and assessed selected chemical and microbiological safety parameters in commercially available products. Lycopene levels were determined spectrophotometrically and by HPLC, whereas pesticide residues, heavy metals, and microbiological purity were analysed using validated regulatory-compliant methods. Marked inconsistencies were found between the declared and measured lycopene content, with HPLC revealing concentrations up to 70% above label claims. Methomyl (0.059 mg/kg), a pesticide not approved in the EU, was detected in one supplement, heavy metal concentrations met current regulatory limits, and other elements remained below quantification thresholds. Microbiological quality was satisfactory, with low total viable counts and absence of pathogens, yeasts, and moulds; only low levels of environmental spore-forming bacteria were detected. The findings highlight acceptable microbiological and elemental safety but reveal substantial deviations in lycopene content labelled/determined and the presence of a non-approved pesticide (however, below the MRL). A comprehensive multi-parameter quality assessment is essential to ensure the safety, reliability, and regulatory compliance of lycopene supplements.

## 1. Introduction

Dietary supplements containing bioactive compounds of plant origin have gained increasing attention worldwide because of their potential health-promoting effects. Among these, lycopene, a naturally occurring carotenoid predominantly found in tomatoes and other red fruits, has been extensively studied for its strong antioxidant activity and possible protective role against chronic diseases [1,2]. Owing to its unique chemical structure, with 11 conjugated double bonds, lycopene acts as a potent quencher of singlet oxygen and a scavenger of free radicals, and is twice as effective as β-carotene [3,4]. These properties have led to its progressive incorporation into dietary supplements, reflecting the growing consumer demand for natural ingredients with proven bioactive potential.

The clinical significance of lycopene has been well documented across various areas of human health. Extensive meta-analyses have shown that lycopene and tomato product supplementation significantly reduce cardiovascular risk factors, particularly by lowering low-density lipoprotein (LDL) cholesterol [5,6]. Furthermore, lycopene has notable antihypertensive effects; lycopene supplementation (especially at doses of 10–15 mg/d) has been shown to significantly reduce systolic blood pressure, particularly in hypertensive individuals. In patients with ischaemic heart failure, lycopene can enhance endothelial function and reduce triglyceride levels, although its effects on other lipid parameters may vary [4,7,8].

Beyond cardiovascular health, lycopene exhibits significant anti-cancer activity by modulating oxidative stress, inducing apoptosis, and inhibiting cell proliferation [9]. Although the impact of lycopene on prostate-specific antigen (PSA) levels remains a subject of debate, some studies have shown no significant reduction in PSA; however, clinical evidence suggests that lycopene may still delay the progression of recurrent prostate cancer and improve treatment outcomes [10]. Additionally, emerging research has highlighted the osteoprotective potential of lycopene, demonstrating that it can preserve bone mineral density and reduce bone resorption markers, making it a promising adjuvant strategy for managing osteometabolic disorders [6,11].

Despite their popularity and documented benefits, dietary supplements represent a heterogeneous category of products that may differ markedly in terms of composition, quality, and safety. Unlike conventional pharmaceuticals, supplements are not subject to stringent regulatory frameworks, which raises concerns about compliance with manufacturers’ declarations and safety standards. Consequently, the evaluation of lycopene-containing supplements should not be limited to the determination of the declared active compound but should also address broader safety parameters.

Microbiological purity is a key quality attribute, as dietary supplements of plant origin may be susceptible to contamination with pathogenic or opportunistic microorganisms [12]. In this context, the Polish Pharmacopeia, in line with international pharmacopeial guidelines, defines specific microbiological quality criteria for herbal preparations and dietary supplements to safeguard public health [13]. Another critical issue is contamination with toxic heavy metals, such as lead, cadmium, arsenic, and mercury [14]. Even at low levels of exposure, these elements can bioaccumulate and exert cumulative toxic effects, contributing to oxidative stress, organ damage, and carcinogenesis [15]. In addition, given that lycopene supplements are derived from plant sources, the presence of pesticide residues cannot be excluded, which further emphasises the need for a systematic safety assessment.

Given the increasing use of lycopene supplements and growing consumer interest in evidence-based quality and safety, comprehensive analytical evaluations are warranted. Therefore, the present study aimed to (i) determine the lycopene content in commercially available supplements, (ii) verify their microbiological safety in accordance with the Polish Pharmacopoeia, and assess contamination with (iii) heavy metals and (iv) pesticide residues. These findings are expected to provide valuable insights into the quality of lycopene-containing supplements and highlight the importance of rigorous monitoring to ensure consumer protection.

This study aimed to evaluate the quality of selected commercially available lycopene dietary supplements by assessing their overall quality, safety, and the presence of selected pesticide residues, heavy metals, and potential pathogenic contaminants.

## 2. Materials and Methods

### 2.1. Samples

Commercial dietary supplements in capsule form containing lycopene were purchased from online stores in Poland in 2025. A total of three different brands were analysed. The tablets were coded (1, 2, 3) to ensure anonymity. Each sample was stored in its original packaging at room temperature and protected from light and humidity until analysis. Capsule contents were as follows (according to the manufacturer’s declaration):

Product/sample no. 1–90 capsules (hypromellose capsule; filler: microcrystalline cellulose; anti-caking agent: magnesium stearate): 10 mg of lycopene in a capsule.

Product/sample no. 2–60 capsules (HPMC-hydroxypropyl methylcellulose): 20 mg of lycopene in a capsule.

Product/sample no. 3–60 capsules (gelatin capsules): 20 mg of lycopene in a capsule.

The powdered material (from the capsules) was homogenised (from 5 to 10 capsules), and representative aliquots were taken for lycopene, heavy metal, pesticide, and microbiological analyses.

### 2.2. Reagents and Standards

All solvents were of an appropriate grade (HPLC, high-performance liquid chromatography; MS, mass spectrometry). Lycopene standard (≥98% purity) was obtained from Sigma-Aldrich (Warsaw, Poland), the metal standard was referenced to NIST, and the confirmation of correctness was NIST SRM 1515; Apple Leaves; National Institute of Standards and Technology; U.S. Department of Commerce: Gaithersburg, MD (14 November 2022) and NIST SRM 1547; Peach Leaves; National Institute of Standards and Technology; U.S. Department of Commerce: Gaithersburg, MD (02 April 2019) with calibration solution of ICP Multi-element standard solution (Merck, Darmstadt, Germany) and pesticide standards from LGC Standards GmbH (Luckenwalde, Germany).

### 2.3. Lycopene Determination

#### 2.3.1. Spectrophotometric Method

Lycopene levels were determined according to the method described by Fish et al. [16]. For each sample, 0.5 g of ground tablet powder (to an accuracy of 0.0001 g) was weighed into a 50 mL centrifuge tube. Lycopene was extracted with hexane:acetone:ethanol, 2:1:1, *v*/*v*/*v*, containing 0.05% BHT to minimise oxidation (ChemPur, Piekary Śląskie, Poland). The mixture was vortexed for 10 min and left for phase division. Absorbance was measured using a UV–Vis DR 5000, Lange (Berlin, Germany, Hach Lange GmbH), at two selected wavelengths, 488 nm and 503 nm, with hexane as the blank. Lycopene concentration was calculated according to the Beer–Lambert law using an extinction coefficient of lycopene and expressed as mg/100 g product. The results were compared with those obtained by HPLC.

#### 2.3.2. HPLC Method

Samples (500 mg) were suspended in 12 mL of water containing butylated hydroxytoluene (BHT, 0.01 g). Samples were shaken in an ultrasonic bath (30 min), then ethanol (16 mL) and dichloromethane (DCM) added to a final volume of 40 mL and stored in the dark for 2 h (chemicals purchased from ChemPur, Piekary Śląskie, Poland). Twenty μL of the supernatant was injected into the HPLC column (Suplex pKb-100 250 × 4.6 mm, 5 μm) (Supelco HPLC Products Analytical, Bellefonte, PA, USA), which was maintained at a temperature of 30 °C. HPLC analysis was performed in accordance with the AOAC method [17], using an HPLC 1260 Infinity with a binary pump and a diode-array detector (DAD detector) (Agilent Technology, Warsow, Poland). Separation was achieved using a Suplex pKb-100 250 × 4.6 mm, 5 μm column (Supelco HPLC Products Analytical, Bellefonte, PA, USA). The mobile phase consisted of methanol with the addition of BHT (0.005% *w*:*v*), 2-propanol (2% *v*:*v*), N-ethyldiisopropylamine (0.02% *v*:*v*), ammonium acetate (2.5% *v*:*v*), and acetonitrile (45.5% *v*:*v*) at a flow rate of 0.8 mL min^−1^ in isocratic flow (chemicals purchased from Chemland, Stargard, Poland). Detection was performed at λ = 488 nm for lycopene, which was identified by an external standard.

### 2.4. Determination of Heavy Metals

Heavy metals (Pb, Cd, As, and Hg) were quantified using inductively coupled plasma mass spectrometry (ICP-MS) and atomic absorption spectrometry (AAS). Calibration curves were established using multi-element standard solutions, and method accuracy was verified using certified reference materials and spiked recovery experiments. The results were expressed in mg/kg of dry weight and compared with the maximum permissible levels established by the European Union for dietary supplements.

The concentrations of cadmium (Cd), lead (Pb), and arsenic (As) were determined using an inductively coupled plasma mass spectrometer (ICP-MS; XSeries 2, Thermo Scientific, Bremen, Germany), following the EN 15763:2009 standard [18]. The samples were digested in 5 mL of 65% HNO_3_ using a microwave digestion system. The resulting solution was quantitatively transferred into a 50 mL volumetric flask and diluted to volume with deionised water.

Mercury (Hg) content was determined by atomic absorption spectrometry (AAS) with an amalgamation technique using an AMA-254 analyser (Altec Ltd., Prague, Czech Republic). This method involves the direct combustion of the sample at 550 °C in an oxygen atmosphere. Mercury concentration was measured by atomic absorption at 254 nm and calculated from the absorbance curve. Certified reference materials (NIST SRM 1515 Apple Leaves and NIST SRM 1547 Peach Leaves, Gaithersburg, MD, USA) were prepared and analysed in the same manner as the samples to ensure analytical accuracy and precision.

### 2.5. Pesticide Determination

#### 2.5.1. Examined Substances

The identified substances were grouped into systematic groups based on their chemical structures and mode of action: insecticides (organochlorine, organophosphate, pyrethroid, carbamate, and others), fungicides (dithiocarbamate, carbamate, and others), herbicides, growth regulators, and others (a list of the pesticides and methods of determination is provided in the Supplementary Tables). All reagents used were purchased at J.T. Baker (Phillipsburg, NJ, USA; Gliwice, Poland).

#### 2.5.2. Methods

All methods used were accredited. The analytical equipment used for the determination of pesticide residues were as follows: gas chromatographs with mass detectors (Agilent Technologies GC 6850 Series + 5973 MSD detector (ditiocarbamates) (Santa Clara, CA, USA, purchased in Poland); Agilent Technologies GC 6890N + 5975B inert XL MSD detector (ethylene oxide) (Wilmington, DE, USA, purchased in Poland); Agilent Technologies GC 7890A + 7000 GC/MS Triple Quad detector (multimethod); Agilent Technologies GC 7890B + 7000D GC/MS Triple Quad detector (multimethod) (both Shanghai, China, purchased in Poland)) and liquid chromatographs with mass detectors (Agilent Technologies HPLC 1200 Series + 6410 Triple Quad LC/MS detector (multimethod); Agilent Technologies HPLC 1260 Infinity + 6460 Triple Quad LC/MS detector (multimethod); Agilent Technologies HPLC 1260 Infinity II + 6470 A Triple Quad LC/MS detector (QuPPe method); Agilent Technologies HPLC 1290 Infinity II + 6470 B Triple Quad LC/TQ detector (QuPPe method) (all Waldbronn, Germany, purchased in Poland)).

#### 2.5.3. PN-EN 15662:2018—GC-MS/MS Technique and LC-MS/MS Technique

This multiresidue method was designed for the extraction and analysis of 499 pesticide residues in samples utilising both gas chromatography–tandem mass spectrometry (GC–MS/MS) and liquid chromatography–tandem mass spectrometry (LC–MS/MS). The procedure follows the QuEChERS (Quick, Easy, Cheap, Effective, Rugged, and Safe) protocol and adheres to the PN-EN 15662:2018 standard [19].

A 10 g sample was weighed into a 50 mL polypropylene Falcon tube, and 10 mL acetonitrile was added. The mixture was vortexed for approximately 3 min. Subsequently, a salt and buffer mixture comprising 4 g anhydrous magnesium sulphate, 1 g sodium chloride, 1 g sodium citrate dihydrate, and 0.5 g sodium hydrogen citrate was introduced, and the tube was shaken for an additional 3 min. After centrifugation at 7200 rpm for 5 min at room temperature, 1 mL of the supernatant was transferred to a 2 mL Eppendorf microcentrifuge tube containing 150 mg anhydrous magnesium sulphate and 25 mg primary-secondary amine sorbent. The tube was vortexed for 1 min and centrifuged at 8500 rpm for 1 min at room temperature. The purified extract was then prepared for analysis using both GC-MS/MS and LC-MS/MS.

For GC–MS/MS, 1 mL of the extract was transferred to an autosampler vial, followed by the addition of 50 µL of internal standard, 100 µL of acetonitrile, and 30 µL of the protector working solution (AP-MIX). The analysis was performed on an Agilent 7890A gas chromatograph with a 7000 Triple Quadrupole mass spectrometer using a DB-5MS capillary column (30.0 m × 250 µm × 0.25 µm) for compound separation. Identification and quantification were conducted using the multiple reaction monitoring (MRM) technique.

For LC-MS/MS, 200 µL of the extract is transferred to a microcentrifuge tube containing 700 µL of solvent A, followed by the addition of 50 µL of the internal standard and 50 µL of acetonitrile. The mixture is filtered into an autosampler vial and analysed using an Agilent 1200 Series liquid chromatograph with a 6410 Triple Quadrupole mass spectrometer. Separation is achieved on an Agilent Eclipse Plus C18 column (2.1 × 100 mm, 1.8 µm). The mobile phases consist of solvent A (water with 5 mM ammonium formate and 0.01% formic acid) and solvent B (acetonitrile: water, 95:5, *v*/*v*, with 5 mM ammonium formate and 0.01% formic acid). Detection is performed using an electrospray ionisation (ESI) source in positive ion mode, operating in dynamic multiple reaction monitoring (DMRM) mode.

The method enables the quantification of all analytes at limits of quantification (LOQ) of 0.01 or 0.005 mg·kg^−1^, as detailed in the Supplementary Tables.

The sample preparation and pesticide extraction procedures were the same as those described above, except for the sample purification step using a primary-secondary amine. Analyses were performed using LC-MS/MS. The range of substances analysed using this method is presented in the Supplementary Tables. All substances analysed by the sub-method 1 were detected at 0.005 mg kg^−1^ LOQ.

##### Sub-Method 2

This method, based on a modification of the PN-EN 15662:2018 standard, incorporates an alkaline hydrolysis step into the QuEChERS extraction procedure for the analysis of acid herbicides [20]. The extracts were analysed using liquid chromatography–tandem mass spectrometry (LC-MS/MS). A 10 g sample was weighed into a 50 mL Teflon tube. To this, 10 mL of HPLC-grade water, 10 mL of acetonitrile, and 1 mL of 5 N sodium hydroxide solution were added. The mixture was shaken for 30 min. Subsequently, 1 mL of 5 N sulfuric acid solution and 100 µL of formic acid were added, and the mixture was shaken for an additional minute. A salt and buffer mixture consisting of 4 g of anhydrous magnesium sulphate, 1 g of sodium chloride, 1 g of sodium citrate dihydrate, and 0.5 g of sodium hydrogen citrate was then added, and the tube was shaken for 1 min. The sample was centrifuged at 8100 rpm for 5 min at room temperature. An aliquot (200 µL) of the extract was transferred to a microcentrifuge tube containing 700 µL of solvent A. Subsequently, 50 µL of the internal standard and 50 µL of acetonitrile were added. The mixture was filtered into an autosampler vial for analysis. The analysis was conducted using an Agilent 1260 Series liquid chromatograph equipped with a 6460 Triple Quadrupole mass spectrometer. Compound separation was achieved using an Agilent Eclipse Plus C18 column (2.1 × 100 mm, 1.8 µm). The mobile phases used were solvent A (water with 0.2% *v/v* acetic acid) and solvent B (acetonitrile). Detection was performed using an electrospray ionisation (ESI) source in both positive and negative ion modes, with multiple reaction monitoring (MRM) for ion detection.

This modified approach allows for the quantification of 299 pesticide residues with a limit of quantification (LOQ) of 0.01 mg·kg^−1^ for all the analysed compounds.

##### PN-EN 15662:2018—LC-MS/MS Technique: Primary-Secondary Amine-Free Variant and Acidic Herbicides

The primary-secondary amine-free variant of the QuEChERS-based method omits the primary-secondary amine cleanup step, which is used to quantify nine selected compounds. The Acidic Herbicide variant of the QuEChERS-based method includes alkaline hydrolysis of samples prior to analysis using LC-MS/MS. It was optimised for determining 33 acidic herbicides (Supplementary Tables).

#### 2.5.4. Dithiocarbamates; Method: PN-EN 12396-2:2002 (Accredited Method)

A gas chromatographic method employing the headspace technique, combined with either flame photometric detection (FPD) or mass spectrometric detection (MS), was used to determine carbon disulfide (CS_2_) released from dithiocarbamate fungicides (such as mancozeb, maneb, propineb, thiram, and zineb) under specific hydrolytic conditions. This approach was adapted from the PN-EN 12396-2:2002 standard, with a modification in which CS_2_ was adsorbed into an isooctane layer [21]. In the procedure, 50.0 g of a homogenised sample was weighed into a cleavage vessel, followed by the addition of 10 mL of isooctane. Subsequently, 75 mL of hydrolysis reagent (0.066 M tin (II) chloride in 4 M hydrochloric acid) and 100 µL of internal standard working solution (100 µg/mL) were added. The vessel was immediately sealed with a screw cap fitted with a septum. The samples were incubated in a shaking water bath at 85 °C for 3 h. Within 2–5 min of immersion, the screw cap was further tightened, and after 10–20 min, the vessels were shaken, particularly those showing signs of coagulation. The samples were shaken every ~60 min again to ensure that any material adhering to the cap was washed down. After 3 h, the vessels were cooled to <10 °C (or at least <20 °C) in a cooling water bath. Before opening, the vessels were shaken to ensure complete absorption of CS_2_ from the headspace into the isooctane phase. An aliquot of the isooctane layer was then transferred to GC vials, with the volume adjusted to nearly fill the vial (e.g., 1.5 mL of isooctane extract in a well-sealed vial) to minimise the headspace. The isooctane extract was analysed directly using an Agilent 6850 Series GC system coupled with a 5973 N mass selective detector for qualitative and quantitative CS_2_ analysis. This method achieved a limit of quantification (LOQ) of 0.01 mg·kg^−1^ for total dithiocarbamate residues.

#### 2.5.5. “QuPPe” Method

The QuPPe (Quick Polar Pesticides) method is a set of analytical protocols developed for the detection and quantification of highly polar pesticides in plant-based food matrices. The process begins with simultaneous extraction using acidified methanol, followed by analysis via liquid chromatography–tandem mass spectrometry (LC-MS/MS) on an Agilent 1200 Series HPLC system with a 6460 Triple Quadrupole detector. For sample preparation, the test portion is adjusted for water content and extracted with the acidified methanol. In the case of cereals, pulses, nuts, and oilseeds, ethylenediaminetetraacetic acid (EDTA) is added to bind metal ions such as calcium and magnesium which can interfere with the analysis of certain compounds, such as glyphosate and AMPA. The extract is then centrifuged, filtered, and subjected to direct LC-MS/MS analysis. To ensure accuracy, isotope-labelled internal standards of the target analytes are introduced at the start of the procedure. These standards account for potential variability arising from volume inconsistencies, analyte loss during preparation, and matrix effects during measurement.

The QuPPe-PO method enables the quantification of all analytes at a limit of quantification (LOQ) of 0.01 mg·kg^−1^. All results were calculated in compliance with the SANTE/10704/2021 guidelines.

### 2.6. Microbiological Quality Testing

Microbiological testing was performed in accordance with the European Pharmacopeia (11th ed.) and the acceptance criteria specified in Chapter 5.1.4 for non-sterile oral products [13]. Samples were aseptically suspended in buffered peptone water (BPW) at a ratio of 1:10 (*w*/*v*) and homogenised to obtain the initial dilution. Serial ten-fold dilutions were prepared and used for subsequent microbiological analyses. The total aerobic microbial count (TAMC) was determined on plate count agar after incubation at 30–35 °C for 3–5 d, whereas the total yeast and mould count (TYMC) was assessed on Sabouraud dextrose agar incubated at 20–25 °C for 5–7 d. Results were expressed as colony-forming units per gram of product (CFU/g).

The presence of *Escherichia coli* was evaluated by selective plating on MacConkey agar after incubation at 30–35 °C for 18–24 h. The detection of *Salmonella* spp. was performed using a standard enrichment procedure, including non-selective enrichment in BPW, selective enrichment in a Rappaport–Vassiliadis broth, and subsequent plating on XLD agar. Results were reported as presence or absence in the tested sample mass.

Representative colonies recovered from enumeration plates were subcultured and identified using matrix-assisted laser desorption/ionisation time-of-flight mass spectrometry (MALDI-TOF MS—MALDI Biotyper, Bruker Daltonics, Bremen, Germany) with a Bruker Microflex system (Bruker Daltonics, Bremen, Germany). Identification scores ≥ 2.0 were considered dependable at the species level, whereas scores between 1.7 and 1.99 were interpreted as genus-level identifications.

Microbiological quality was evaluated according to the acceptance criteria defined in the European Pharmacopeia (Ph. Eur. Eur. 5.1.4) for non-sterile oral preparations.

All culture media were purchased from Oxoid (Thermo Fisher Scientific, Basingstoke, UK), unless otherwise stated.

### 2.7. Statistical Analysis

Statistical analyses were performed using STATISTICA v.13 (Dell Inc., 2016, Tulsa, OK, USA). A one-way analysis of variance (ANOVA) was used to assess differences between samples. Significant differences among means were determined at *p* = 0.05 using Tukey’s HSD test. All results are expressed as means ± standard deviation from three replicates.

## 3. Results

### 3.1. Lycopene Content

The lycopene content declared by the producers differed from the determined values. In the case of the spectrophotometer method, the values at both wavelengths were similar. However, the value obtained at 503 nm was lower than that at 448 nm. The values obtained from HPLC determination were higher than those declared by producers (5%, 70.39%, and 60.16% for samples 1, 2, and 3, respectively).

**Figure 1:**
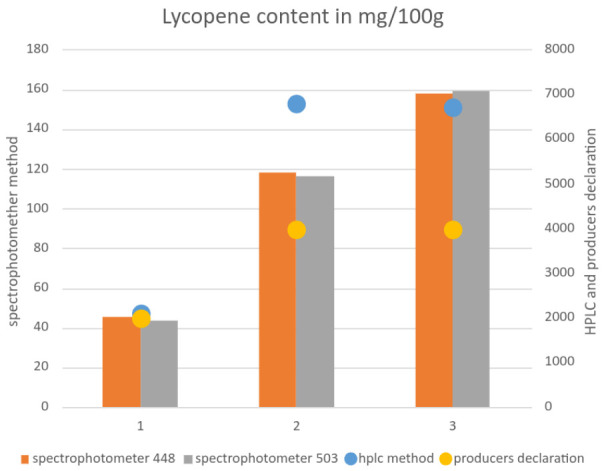
Lycopene content in supplements compared: label declaration, HPLS and spectrophotometric method.

The results of lycopene determination using a spectrophotometer at different wavelengths were similar, with differences between wavelengths ranging from 0.68% to 4.48%. The values obtained by HPLC were significantly higher (Table 1, Figure 1). Moreover, for sample 1, the values declared by the manufacturer were consistent with those determined experimentally; for the remaining samples, the measured lycopene content was approximately 70% higher than the declared value.

The discrepancies between the spectrophotometric and HPLC methods of lycopene determination in dietary supplements (mainly for sample no. 2) may be attributed to matrix effects. Spectrophotometric methods, while rapid and cost-effective, are more susceptible to interference from co-extracted compounds, excipients, and capsule materials that may absorb at similar wavelengths or influence extraction efficiency. In contrast, HPLC provides higher selectivity and allows the separation of lycopene from other carotenoids, degradation products, and matrix constituents, resulting in more specific quantification. The largest differences between the two analytical approaches were observed for sample no. 2. This may be explained by differences in composition, polymer structure, and potential interactions between lycopene and filler materials, which could affect extraction recovery and spectral interference. In contrast, the smallest discrepancy was noted for sample nos. 1 and 3. The more complex but stable excipient matrix, together with the lower declared lycopene content, may have resulted in reduced analytical bias between the two methods. These findings highlight the importance of considering formulation-specific matrix effects when selecting and validating analytical procedures for carotenoid quantification in dietary supplements.

### 3.2. Lycopene Supplement Safety

#### 3.2.1. Pesticide Presence

In an analysis conducted on only one sample—no. 3—there were pesticides determined: a carbamide insecticide—metomyl (0.059 mg/kg)—that did not exceed MRLs, with ADI 0.0025 mg/kg bw/d. However, this substance is not approved for use in the EU (expiration of approval: 31 August 2019).

#### 3.2.2. Heavy Metal Content

In the analysis of the tested supplements, no exceedances of heavy metal limits were identified with respect to Commission Regulation (EU) 2023/915 (Table 2) [21].

A conformity assessment was performed in accordance with the ILAC-G8:09/2019 guideline using a simple binary decision rule. Cadmium (Cd) and lead (Pb) concentrations complied with the applicable maximum levels established under current EU legislation, as the measured values were below the specified tolerance limits.

For arsenic (As), chromium (Cr), nickel (Ni), and mercury (Hg), no specific maximum levels (MLs) are defined for dietary supplements under current EU legislation. Therefore, formal conformity assessment could not be applied. Nevertheless, the measured concentrations were low and within the ranges typically reported for plant-derived products in the scientific literature.

#### 3.2.3. Microbiological Safety

The microbiological quality of three lycopene-containing dietary supplements (1, 2, and 3) was assessed in accordance with the European Pharmacopoeia methods. All analysed products showed low levels of microbial contamination. The total aerobic microbial count (TAMC) ranged from 0.6 × 10^3^ to 0.7 × 10^3^ colony-forming units (CFU)/g, while the total yeast and mould count (TYMC) was below the detection limit in all samples. *Escherichia coli* was not detected in 1 g of any product, and *Salmonella* spp. were absent in 10 g of all tested samples, indicating compliance with the acceptance criteria for non-sterile oral preparations.

The use of MALDI-TOF MS for species-level identification provides a higher-resolution assessment of microbiological quality than routine enumeration alone and enables differentiation between clinically relevant contaminants and environmental microbiota with negligible health risks.

Representative colonies recovered from enumeration plates were subjected to identification by MALDI-TOF MS. Microorganisms were detected in all analysed supplements, although their qualitative composition differed between products. In sample no. 1, MALDI-TOF MS identified *Paenibacillus simplex*, *Paenibacillus pabuli*, *Priestia megaterium* (formerly *Bacillus megaterium*), *Bacillus altitudinis/pumilus*, *Alkalihalobacillus gibsonii*, and *Staphylococcus hominis*. In sample no. 2 *Priestia megaterium* and *Paenibacillus pabuli* were detected, whereas supplement no. 3 contained *Paenibacillus simplex* and *Priestia megaterium*.

Overall, the analysed lycopene-containing dietary supplements demonstrated satisfactory microbiological quality. All products exhibited low total microbial counts and the absence of the specified microorganisms, consistent with previous reports indicating that properly manufactured dietary supplements generally meet microbiological safety requirements, despite being non-sterile products (Table 3, Table 4, Table 5 and Table 6).

## 4. Discussion

The determination of lycopene content in dietary supplements revealed discrepancies between the values declared by the manufacturers and the experimentally obtained results. Interestingly, while spectrophotometric measurements at 448 nm and 503 nm yielded relatively consistent results, HPLC analysis showed significantly higher lycopene concentrations (up to 70% higher than those reported for samples 2 and 3). A comparison was performed between HPLC and spectrophotometric methods because of the widespread use of both techniques; the former is valued for its accuracy and precision, and the latter for its speed and analytical simplicity. The results obtained for the three different matrices varied substantially, which may indicate a strong matrix effect influencing the measurements (including capsule composition and excipient interactions that influence extraction efficiency and analytical selectivity). The largest differences were observed in HPMC, whereas the smallest discrepancy occurred in the hypromellose-based products with microcrystalline cellulose, magnesium stearate, and gelatin capsule formulations, underscoring the critical role of formulation characteristics in method-dependent variability. These differences highlight the critical importance of selecting appropriate analytical methods for quality control. HPLC is generally regarded as the gold standard for carotenoid analysis because of its ability to separate lycopene from other matrix components and isomers [1]. The higher values detected by HPLC suggest that spectrophotometric methods may underrepresent the total lycopene content, possibly due to interference or the presence of various cis isomers, which exhibit different absorption properties than all transforms [2,3,23]. Moreover, as noted in the literature, the accumulation and bioavailability of lycopene in supplements may depend on its composition, with products containing fats having a beneficial effect on lycopene absorption [24].

Pesticide residue analysis identified the presence of methomyl (0.059 mg/kg) in one of the tested samples. Although this concentration did not exceed the general maximum residue levels (MRLs), it is crucial to note that methomyl is no longer approved for use in the European Union. This finding underscores a significant gap in the regulatory oversight of dietary supplements, which are not subject to the same stringent safety frameworks as conventional pharmaceuticals. The presence of substances such as pesticides, even at low levels, is concerning because they are known to induce oxidative stress and genotoxicity in human cells [4,6]. However, experimental evidence suggests that lycopene acts as a potent protective agent against pesticide-induced toxicity. Studies have demonstrated that lycopene can reduce the cytotoxic and genotoxic effects of various insecticides (e.g., dimethoate and lambda-cyhalothrin) in human lymphocytes by scavenging free radicals and enhancing antioxidant enzyme activities [4,25].

The assessment of heavy metal content in the analysed food supplements demonstrated that cadmium (Cd) and lead (Pb) concentrations were within the maximum levels established by Commission Regulation (EU) 2023/915 [21].

In contrast, arsenic (As), chromium (Cr), nickel (Ni), and mercury (Hg) levels were below the limit of quantification or detected at levels that did not allow for a clear assessment of regulatory compliance. An important fact is that lycopene plays a vital role in mitigating the harmful effects of these metals. For instance, lycopene has been shown to normalise oxidative stress biomarkers (MDA, SOD, and CAT) and protect against DNA fragmentation induced by heavy metal pollution [25,26,27]. Its ability to function as a potential chelating and anti-apoptotic agent provides a secondary layer of protection for consumers [25,28,29].

The present study demonstrates that the analysed lycopene-containing dietary supplements exhibited satisfactory microbiological quality. The characteristics of the microorganisms identified in the analysed supplements, together with their potential health relevance to consumers, are summarised in Table 7.

All products showed low levels of microbial contamination and the absence of yeasts, moulds, and specified pathogens, which is consistent with other reports indicating that properly manufactured dietary supplements generally meet microbiological safety expectations, despite being non-sterile products [30,34].

The detected microorganisms belonged predominantly to environmental spore-forming bacteria, including representatives of the genera *Bacillus*, *Paenibacillus*, *Priestia*, and *Alkalihalobacillus*. Such microorganisms are commonly reported in dietary supplements, particularly those containing plant-derived ingredients, because of their natural occurrence in soil and botanical raw materials and their ability to survive adverse environmental conditions associated with drying and processing [30,32,34].

Among the identified taxa, *Bacillus altitudinis*/*pumilus* and *Priestia megaterium* are generally regarded as low-risk environmental microorganisms, although sporadic opportunistic infections have been reported, primarily in immunocompromised individuals [31,34,35]. Importantly, the risk associated with *Bacillus pumilus* is considered substantially lower than that associated with toxigenic *Bacillus cereus* and is mainly of clinical relevance in vulnerable populations. Similarly, *Paenibacillus simplex* and *Panbacillus pabuli* are infrequently associated with human disease and are more commonly interpreted as indicators of environmental or raw material contamination rather than direct health hazards in orally administered products [31,35].

The occasional detection of *Staphylococcus hominis*, a coagulase-negative *Staphylococcus* and common human skin commensal, is consistent with incidental contamination related to handling or the production environment. Coagulase-negative *staphylococci* are frequently reported in food and pharmaceutical settings and are generally considered to pose a negligible risk in oral products when present at low levels and in the absence of specific virulence determinants [37].

The high concentration of lycopene found in the tested supplements (particularly those exceeding label claims) has direct implications for their therapeutic efficacy. Lycopene is recognised as one of the most potent natural antioxidants, with a singlet-oxygen-quenching capacity twice that of β-carotene [1,6]. Clinical studies have confirmed its benefits in reducing cardiovascular risk factors, such as by lowering low-density lipoprotein cholesterol and improving endothelial function [2,4]. Moreover, lycopene supplementation is increasingly being investigated for its role in bone health, where it stimulates osteoblastic activity and inhibits bone resorption, potentially offering an adjuvant strategy for managing osteoporosis [6]. Given its dose-dependent effects in areas such as prostate cancer prevention and blood pressure regulation, ensuring accurate labelling and microbiological and chemical purity is essential for consumer safety and the achievement of intended health outcomes [1,3].

### Limitations of the Study

The main limitation of this study is the small sample size, as the analysis was conducted on only three samples, which prevents generalisation of the results to the broader market. The limited sample size reflects the exploratory nature of the study and the practical limitations of sampling and analytical procedures. However, the depth and rigour of the analytical methods applied to each individual sample ensure the reliability of the results obtained within this limited data set. Although the results cannot be considered representative of the entire market, they do have significant warning value, pointing to potential issues that require further, larger-scale research.

## 5. Conclusions

The present study shows that the quality of commercially available lycopene-containing dietary supplements is variable. Considerable discrepancies were found between the declared and experimentally determined lycopene levels, with HPLC measurements reaching up to 70% above label claims, as spectrophotometric methods may underestimate lycopene content due to matrix effects and the presence of lycopene forms. Although only three supplements were examined—limiting the ability to draw broad market-wide conclusions—the observed inconsistencies highlight issues that may reflect wider trends relevant to consumers.

The detection of methomyl, despite concentrations below general MRL thresholds, raises concerns regarding regulatory compliance and supply-chain oversight, particularly given that this pesticide is no longer approved for use in the European Union. This finding underscores the need for stricter monitoring of raw materials used in supplement production. Regarding toxic elements, cadmium and lead levels met current European standards, and other monitored metals remained below the limit of quantification. Microbiological assessment confirmed satisfactory safety across all tested products. Low total microbial counts, absence of pathogens, and the predominance of environmental spore-forming bacteria are consistent with the expected microbiological profile of properly manufactured plant-based supplements. The identified taxa—mainly *Bacillus*-related species and occasional coagulase-negative *staphylococci*—pose minimal risk at the observed levels and indicate appropriate hygienic practices during production and storage.

Overall, this study emphasises the importance of a comprehensive quality evaluation of dietary supplements, including accurate quantification of active ingredients, monitoring of pesticide residues and heavy metals, and microbiological safety assessment. Although the sample size was limited, the findings provide valuable insights for consumers and suggest potential quality-related challenges that may extend beyond the tested products. Ensuring analytical precision, regulatory compliance, and chemical and microbiological purity remains essential for both therapeutic efficacy and consumer protection.

## Figures and Tables

**Table 1:** Lycopene content determined according to the method used.

	Spectrophotometer’s Measurement at:				HPLC	
	448 nm	SD	503 nm	SD	488 nm	SD
	mg/100 g					
Sample 1	45.27	±0.83	43.24	±4.18	2115.26	±10.87
Sample 2	118.28	±0.07	116.19	±3.55	6815.45	±4.18
Sample 3	158.03	±2.04	159.10	±0.23	6726.35	±12.88

**Table 2:** Metal content in lycopene-containing dietary supplements.

Metal Content (mg/kg)	Sample No.			
	1	2	3	MRL/ML
As	0.002 ± 0.001	0.007 ± 0.001	0.025 ± 0.004	nd
Cd	0.004 ± 0.001	0.013 ± 0.003	0.007 ± 0.001	1
Ni	0.067 ± 0.013	1.25 ± 0.25	0.88 ± 0.17	nd
Cr	0.010 ± 0.002	0.93 ± 0.26	0.61 ± 0.17	nd
Pb	0.007 ± 0.001	0.016 ± 0.003	0.093 ± 0.018	3
Hg	<LOQ	<LOQ	0.0014 ± 0.001	nd

Note: nd—not defined; ML (maximum level)/MRL (maximum residue level) in accordance with Commission Regulation (EU) 2023/915 of 25 April 2023 and Commission Regulation (EU) 2018/73 on maximum residue levels for mercury compounds [22]. LOQ for mercury determination is 0.001 mg/kg.

**Table 3:** Microbiological quality of lycopene dietary supplement no. 1.

Parameter	Result	Acceptance Criterion (Ph. Eur. 5.1.4)
TAMC	0.7 × 10^3^ CFU/g	≤10^4^ CFU/g
TYMC	0 CFU/g	≤10^2^ CFU/g
*Escherichia coli*	Not detected	Absent in 1 g
*Salmonella* spp. (10 g)	Not detected	Absent in 10 g

**Table 4:** Microbiological quality of lycopene dietary supplement no. 2.

Parameter	Result	Acceptance Criterion (Ph. Eur. 5.1.4)
TAMC	0.6 × 10^3^ CFU/g	≤10^4^ CFU/g
TYMC	0 CFU/g	≤10^2^ CFU/g
*Escherichia coli*	Not detected	Absent in 1 g
*Salmonella* spp. (10 g)	Not detected	Absent in 10 g

**Table 5:** Microbiological quality of lycopene dietary supplement no. 3.

Parameter	Result	Acceptance Criterion (Ph. Eur. 5.1.4)
TAMC	0.7 × 10^3^ CFU/g	≤10^4^ CFU/g
TYMC	0 CFU/g	≤10^2^ CFU/g
*Escherichia coli*	Not detected	Absent in 1 g
*Salmonella* spp. (10 g)	Not detected	Absent in 10 g

**Table 6:** Identified microbial contaminants in the analysed lycopene dietary supplements (MALDI-TOF MS).

Sample No.	Identified Microorganisms
1	*Paenibacillus simplex*, *Paenibacillus pabuli*, *Priestia megaterium*, *Bacillus altitudinis*/*pumilus*, *Alkalihalobacillus gibsonii*, *Staphylococcus hominis*
2	*Priestia megaterium*, *Paenibacillus pabuli*
3	*Paenibacillus simplex*, *Priestia megaterium*

**Table 7:** Microorganisms identified in the study and the interpretation of occurrence.

Microorganism Identified in This Study	Reported Frequency in Dietary Supplements *	Typical Origin	Interpretation	Potential Risk for Consumers	Ref.
*Bacillus altitudinis/Bacillus pumilus*	20–60%	Soil/plant raw material	Typical spore-formers in plant supplements	Negligible	[30,31,32,33,34]
*Priestia megaterium* (formerly *Bacillus megaterium*)	20–60%	Environmental	Raw material microbiota	Negligible	[30,31,32]
*Paenibacillus simplex*	<20%	Soil	Sporadic environmental contaminant	Very low	[31,35,36,37]
*Paenibacillus pabuli*	Rare (<10%)	Plant material	Environmental contamination	Very low	[35,36,37]
*Alkalihalobacillus gibsonii*	Rare (<10%)	Environmental	Background environmental spore-forming microbiota	Negligible	[32,36]
*Staphylococcus hominis*	5–25%	Human skin	Handling/environmental contamination	Low (only in immunocompromised cases)	[30,34,38,39]

* Reported frequencies primarily refer to genus-level detection in dietary supplements; species-level prevalence data are rarely provided in the literature.

## Data Availability

The original contributions presented in this study are included in the article/Supplementary Materials. Further inquiries can be directed to the corresponding author.

## Supplementary Materials

The following supporting information can be downloaded at: https://www.mdpi.com/article/10.3390/foods15091583/s1, Table S1. Description of analytical method according product, Table S2. Method description according to determined substance (and LOQ) in certain product. Table S3. Methods details. Table S4. Multi LC method.
