# Supplementary material for: Preliminary Quality and Safety Evaluation of Lycopene-Based Dietary Supplements: Analysis of Active Compound Content, Microbiological Purity, and Chemical Contaminants
Source: Foods. 2026 May 4;15(9):1583. doi: 10.3390/foods15091583 (PMC13164322; doi:10.3390/foods15091583)
Supplement: Supplementary file 1 [file foods-15-01583-s001.zip › foods-4221286-supplementary.pdf]

Tab.S1. Description of analytical method according product

Food, agricultural products, plant material		
Product description	Analitycal method	Determined substances (method and LOQ given in the table 3)
specific or unique products<br>(SANTE group 6)<br><br>e.g.:<br>- hops<br>- cocoa beans and related products<br>- coffee<br>- tea<br>- spices<br>- dried herbs	PN-EN 15662:2018	2,4-D, 2,4-DB, 2,4,5-T, DDD-o,p’, DDD-p,p’, DDE-o,p’, DDE-p,p’, DDM, DDT-o,p’, DDT-p,p’, DDAC C8, BAC C10, BAC C8, abamectin, acephate, acetamiprid, acetochlor, aclonifen, acrinathrin, aldrin, aldicarb, aldicarb sulfone, aldicarb sulfoxide, allethrin, amemetocradin, amidosulfuron, amisulbrom, aminocarb, ametryn, anthraquinone, azadirachtin, azaconazole, azinphos ethyl, azinphos-methyl, aziprotryne, azoxystrobin, bendiocarb, benalaxyl, benfluralin, benfuracarb, beflubutamid, bifenazate, bifenazate diazene, bifenox, bifenthrin, biphenyl, bitertanol, bixafen, boscalid, bromacil, bromocyclen, bromfenvinphos, bromophos, bromophos-ethyl, bromopropylate, bromuconazole, bupirimate, buprofezin, butachlor, butafenacil, butylate, cadusafos, carbaryl, carbendazim, carbetamide, carbofuran, carbofuran 3-hydroxy, carbofuran 3-keto, carfentrazone-ethyl, captan, captafol, chlorantraniliprole, chlorbenside, chlorbufam, chlordane-cis, chlordane-trans, chlorfenapyr, chlofenson, chlorfenvinphos, chlormephos, chlorobenzilate, chloropropylate, chlorothalonil, chlorpropham, chlorpyrifos, chlorpyrifos-methyl, chlorthal-dimethyl, chlorthion, chlorthiophos, clodinafop propargyl, clomazone, clofentezine, clothianidin, coumaphos, crimidine, cyanophenphos, cyanophos, cyantraniliprole, cycloate, cyhexatin, cyfluthrin, cyflufenamid, cyflumetofen, cypermethrin, cyprazine, cyproconazole, cyprodinil, deet, deltamethrin, demeton-s, demeton-S-methyl, demeton-S-methyl sulphone, demeton-S-methyl sulphoxide, desmetryn, dialifos, diazinon, dicamba, dichlobenil, dichlofenthion, dichlofluanid, dichlorobenzamide 2,6-, dichlorobenzophenone-p,p, dichlorprop, dichlorvos, diclobutrazol, dicloran, diclofop, dicofol, dieldrin, diethofencarb, difenoconazole, diflufenzopyr, dimethachlor, dimethenamid, dimethoate, dimethomorph, dimoxystrobin, diniconazole, dinitramine, dinobuton, dinoseb, dioxabenzofos, dioxacarb, dioxathion, diphenylamine, disulfoton, disulfoton sulfon, disulfoton sulfoxide, ditalimfos, dodine, dmst, DMF, DMPF, edifenphos, emamectin B1a, emamectin B1b, endosulfan alpha, endosulfan beta, endosulfan sulphate, endrin, endrin keton, epn, epoxiconazole, esfenvalerate, etaconazole, ethalfluralin, ethion, ethofumesate, ethofumesate-2-keto, ethoprophos, ethoxyquin, etofenprox, etrimfos, famoxadone, fenamidone, fenamiphos, fenamiphos sulfoxide, fenamiphos sulphone, fenarimol, fenazaquin, fenbuconazole, fenchlorphos, fenbutatin oxide, fenfuram, fenhexamid, fenitrothion, fenobucarb, fenoxaprop-P-ethyl, fenpropathrin, fenpropidin, fenpropimorph, fenpyrazamine, fensulfothion, fensulfothion oxon, fensulfothion oxon sulphone, fensulfothion sulphone, fenthion, fenthion oxon, fenthion oxon sulphone, fenthion sulfoxide, fenthion sulphone, fipronil, fipronil desulfinyl, fipronil sulfon, fluchloralin, flonicamid, florasulam, flufenacet, flufenoxuron, fluopicolide, fluopyram, fluoxastrobin, flupyradifurone, fluquinconazole, flurochloridone, flurtamone, flutianil, flutolanil, flutriafol, fluxapyroxad, folpet, foramsulfuron, formetanate, formothion, fosthiazate, fuberidazole, furalaxyl, furathiocarb, gamma-cyhalothrin, halfenprox, heptachlor, heptachlor cis-epoxid, heptachlor trans-epoxid, heptenophos, hexachlorobenzene, hexachlorocyclohexane (HCH) alpha, hexachlorocyclohexane (HCH) beta, hexaconazole, imazalil, imazamox, imazapyr, imazethapyr, imidacloprid, indoxacarb, iodofenphos, ipconazole, iprobenfos, iprovalicarb, iprodione, isocarbophos, isofenphos, isofenphos-methyl, isofetamid, isoprocarb, isoprothiolane, isoproturon, isopyrazam, isoxaben, isoxaflutole, isoxathion, kresoxim-methyl, lambda-cyhalothrin, lindane, malathion, mandestrobin, mecarbam, mepanipyrim, mepronil, metalaxyl, metaconazole, metamitron, metazachlor, methacrifos, methidathion, methiamyl, methiocarb, methiocarb sulphone, methiocarb sulphoxide, methomyl, methoprotryne, methoxychlor, metobromuron, metolachlor, metolachlor-s, metosulam, metoxuron, metrafenone, metribuzin, metsulfuron-methyl, mevinphos, molinate, monocrotophos, monuron, myclobutanil, napropamide, nitralin, nitrapyrin, nitrofen, nitrothal isopropyl, nuarimol, omethoate, oxadiazon, oxadixyl, oxamyl, oxycarboxin, paclobutrazol, paraoxon-methyl, parathion, parathion-methyl, penconazole, pencycuron, pendimethalin, penflufen, pentachloroaniline, permethrin, perthane (ethylan), pethoxamid, phenmedipham, phenthoate, phorate, phorate sulfone, phorate sulfoxide, phosalone, phosetyl, phosmet, phosmet oxon, phosphamidon, phthalimide, picolinafen, picoxystrobin, piperonyl butoxide, piperophos, pirimicarb, pirimicarb desmethyl, pirimiphos-ethyl, pirimiphos-methyl, procymidone, profenofos, profluralin, prometryn, prometon, propachlor, propargite, propazine, propetamphos, propham, propiconazole, propyzamide, prothioconazole destio, prothiofos, pyraclostrobin, pyrazophos, pyrethrins, pyridaben, pyridalyl, pyridaphenthion, pyrifenox, pyrimethanil, pyriofenone, pyriproxyfen, pyroquilon, pyroxsulam, quinalphos, quinoxyfen, quintozene, resmethrin, silafluofen, simazine, spiromesifen, spinetoram C42, spinetoram C43, spinosyn a, spinosyn d, spirodiclofen, spirotetramat, spirotetramat enol, spirotetramat enol-glucoside, spirotetramat ketohydroxy, spirotetramat monohydroxy, spiroxamine, sulfotep, sulfosulfuron, sulfoxaflor, tau-fluvalinate, tebuconazole, tebufenozide, tebufenpyrad, tecnazene, tefluthrin, terbacil, terbufos, terbufos oxon, terbufos sulphone, terbufos sulphoxide, terbuthylazine, thiabendazole, thiacloprid, thiamethoxam, thiencarbazone-methyl, thifensulfuron-methyl, thiodicarb, thiometon, thiophanate-methyl, tolclofos-methyl, tolylfluanid, tri-allate, triadimefon, triadimenol, triazophos, trifloxystrobin, triflumizole, trifluralin, triticonazole, tritosulfuron, vinclozolin, zoxamide, TFNA, TFNA-AM, TFNG
	PN-EN 12396-2:2002	dithiocarbamates expressed as cs_2 (table 3)
	PB-04 ed.1 of 10.01.2022	ethylene oxide (2-chloroethanol expressed as ethylene oxide) (table 3)
	EURL-SRM QuPPe - PO	MPA (not acreditated), chlorates, chlormequat chloride, cyromazine, ethephon, glyphosate, glufosinate together with matabolites (MPP, N-acetyl-glufosinate), maleic hydrazide, malamine, matrine, mepiquat chloride, oxymatrine, perchlorates, phosetyl, phosphonic acid, trimethylsulfonyl cation (table 3)

Tab.S2. Method description according to determined substance (and LOQ) in certain product.

Methods	Substances in: SANTE: group 6<br>e.g. cocoa, teas, spices, dried herbs, etc.)	LOQ [mg/kg]
GC-MS/MS	pyrethrins	0.1
	acetochlor, acrinathrin, aldrin, allethrin, ametryn, aminocarb, anthraquinone, atrazine, azaconazole, azinphos ethyl, azinphos-methyl, azoxystrobin, beflubutamid, benalaxyl, benfluralin, benfuracarb, bifenazate, bifenazate diazene, bifenox, bifenthrin, bitertanol, boscalid, bromfenvinphos, bromocyclen, bromophos, bromophos-ethyl, bromopropylate, bupirimate, buprofezin, butachlor, butafenacil, butylate, captan, carbaryl, carboxin, chinomethionat, chlorbenside, chlorbufam, chlordane. -cis, chlordane. -trans, chlorfenapyr, chlorfenson, chlorfenvinphos, chlormephos, chlorobenzilate, chloropropylate, chlorothalonil, chlorpropham, chlorpyrifos, chlorpyrifos-methyl, chlorthal-dimethyl, chlorthion, chlorthiophos, clodinafop propargyl, clomazone, coumaphos, crimidine, cyanophenphos, cyanophos, cycloate, cyfluthrin, cypermethrin, cyprazine, cyproconazole, cyprodinil, DDD-o,p’, DDD-p,p’, DDE-o,p’, DDE-p,p’, DDM, DDT-o,p’, DDT-p,p’, deltamethrin, demeton-S, desmetryn, dialifos, diazinon, dichlobenil, dichlofenthion, dichlofluanid, dichlorobenzamide, 2,6-, dichlorobenzophenone-p,p, dichlorvos, diclobutrazol, dicloran, dicofol, dieldrin, diethofencarb, difenoconazole, dimethachlor, dimethoate, dimethomorph, dimoxystrobin, diniconazole, dinitramine, dinobuton, dinoseb, dioxabenzofos, diphenylamine, disulfoton, ditalimfos, edifenphos, endosulfan alpha, endosulfan beta, endosulfan sulphate, endrin, endrin keton, epn, epoxiconazole, esfenvalerate, etaconazole, ethalfluralin, ethion, ethofumesate, ethofumesate, -2-keto, ethoprophos, ethoxyquin, etofenprox, etrimfos, fenamiphos, fenarimol, fenazaquin, fenbuconazole, fenchlorphos, fenhexamid, fenitrothion, fenpropathrin, fenpropidin, fenpropimorph, fenpyrazamine, fenthion, fenvalerate, fipronil, fipronil desulfinyl, fipronil sulfon, fluchloralin, flucythrinate, fludioxonil, fluensulfone, flumetralin, fluorodifen, fluquinconazole, flurprimidol, flurtamone, flusilazole, flutianil, flutriafol, folpet, formothion, furalaxyl, furathiocarb, gamma-cyhalothrin, halfenprox, heptachlor, heptachlor cis-epoxid, heptachlor trans-epoxid, heptenophos, hexachlorobenzene, hexachlorocyclohexane (HCH) alpha, hexachlorocyclohexane (HCH) beta, hexaconazole, imazalil, iodofenphos, iprobenfos, iprodione, isocarbophos, isofenphos, isofenphos-methyl, isofetamid, kresoxim-methyl, lambda-cyhalothrin, lindane, malathion, mandestrobin, mecarbam, mepanipyrim, mepronil, metalaxyl, metazachlor, metconazole, methacrifos, methidathion, methoxychlor, metolachlor, metribuzin, mevinphos, molinate, myclobutanil, nitralin, nitrapyrin, nitrofen, nitrothal isopropyl, nuarimol, oxadiazon, oxadixyl, oxyfluorfen, paclobutrazol, parathion, parathion-methyl, penconazole, pencycuron, pendimethalin, penflufen, pentachloroaniline, permethrin, perthane (ethylan), pethoxamid, phenthoate, phorate, phorate sulfone, phorate sulfoxide, phosalone, phosmet, phthalimide, picolinafen, picoxystrobin, piperonyl butoxide, piperophos, pirimicarb, pirimicarb desmethyl, pirimiphos-ethyl, pirimiphos-methyl, procymidone, profenofos, profluralin, prometon, prometryn, propachlor, propargite, propazine, propetamphos, propham, propiconazole, propyzamide, prothioconazole destio, prothiofos, pyraclostrobin, pyrazophos, pyridaben, pyridalyl, pyridaphenthion, pyrifenox, pyrimethanil, pyriofenone, pyriproxyfen, quinalphos, quinoxyfen, quintozene, silafluofen, simazine, spiromesifen, sulfotep, tau-fluvalinate, tebuconazole, tebufenpyrad, tecnazene, tefluthrin, terbacil, terbufos, terbutryn, tetrachlorvinphos, tetraconazole, tetradifon, tetrahydrophthalimide, tetramethrin, tetrasul, thiobencarb, tolclofos-methyl, tolylfluanid, triadimefon, triadimenol, tri-allate, triazophos, trifloxystrobin, triflumizole, trifluralin, vinclozolin	0.01
LC-MS/MS	phosphonic acid	0.1
	AMPA*, glyphosate, trimethylsulfonyl cation, glufosinate together with metabolites (MPP, N-acetyl-glufosinate), maleic hydrazide	0.05
	abamectin, acephate, acetamiprid, aclonifen, aldicarb, aldicarb sulfone, aldicarb sulfoxide, ametoctradin, amidosulfuron, amisulbrom, azadirachtin, aziprotryne, azoxystrobin, BAC C10, BAC C8, beflubutamid, bendiocarb, benthiavalicarb isopropyl, benzovindiflupyr, bixafen, boscalid, bromacil, bromuconazole, cadusafos, carbaryl, carbendazim, carbetamide, carbofuran, carbofuran 3-hydroxy,	0.01

	carbofuran 3-keto, carfentrazone-ethyl, chlorates, chlorantraniliprole, chloridazon, chlorotoluron, chlorpyrifos, chlorsulfuron, chromafenozide, clodinafop propargyl, clofentezine, clothianidin, cyantraniliprole, cyazofamid, cyflufenamid, cyflumetofen, cymiazol, cymoxanil, cyproconazole, cyromazine,DDAC C8, DEET, demeton-S-methyl, demeton-S-methyl sulphone, demeton-S-methyl sulphoxide, desmedipham, dicrotophos, diethofencarb, diflubenzuron, dimethenamid, dimethoate, dinotefuran, disulfoton, disulfoton sulfon, disulfoton sulfoxide, diuron, DMF, DMPF, emamectin B1a, emamectin B1b, ethametsulfuron-methyl, ethephon, ethiofencarb, ethirimol, etoxazole, famoxadone, fenamidone, fenamiphos, fenamiphos sulfoxide, fenamiphos sulphone, fenbuconazole, fenfuram, fenhexamid, fenobucarb, fenoxaprop-p-ethyl, fenpropidin, fenpropimorph, fenpyroximate, fensulfothion, fensulfothion oxon, fensulfothion oxon sulphone, fensulfothion sulphone, fenthion, fenthion oxon, fenthion oxon sulphone, fenthion sulfoxide, fenthion sulphone, flonicamid, florasulam, flufenacet, flufenoxuron, fluopicolide, fluopyram, fluoxastrobin, flupyradifurone, flurochloridone, flutianil, flutolanil, flutriafol, fluxapyroxad, foramsulfuron, formetanate, fosthiazate, fuberidazole, halofenozide, hexythiazox, imazalil, imidacloprid, indoxacarb, iodosulfuron methyl, ipconazole, iprovalicarb, isofetamid, isoprocarb, isoprothiolane, isoproturon, isopyrazam, isoxaben, isoxaflutole, isoxathion, lenacil, linuron, lufenuron, malaoxon, malathion, mandipropamid, matrine, melamine, metaflumizone, metalaxyl, metamitron, metazachlor, methamidophos, methiocarb, methiocarb sulphone, methiocarb sulphoxide, methomyl, methoprotryne, methoxyfenozide, metobromuron, Metolachlor-S, metosulam, metoxuron, metrafenone, metsulfuron-methyl, monocrotophos, monuron, napropamide, nicosulfuron, nitenpyram, novaluron, omethoate, oxadixyl, oxamyl, oxycarboxin, oxymatrine, paraoxon-methyl, parathion, parathion-methyl, pencycuron, pendimethalin, penthiopyrad, perchlorates, pethoxamid, phenmedipham, phenthoate, phosetyl, phosmet, phosmet oxon, phoxim, pinoxaden, piperonyl butoxide, prochloraz, prochloraz BTS 44595, prochloraz BTS 44596, propamocarb, propaquizafop, propoxur, propoxycarbazone, proquinazid, prosulfocarb, prosulfuron, pyridaben, pyridafol, pyrifenox, pyriproxyfen, pyroquilon, pyroxsulam, quinoclamine, quizalofop-ethyl, rimsulfuron, rotenone, saflufenacil, silthiofam, spinetoram C42, spinetoram C43, spinosyn A, spinosyn D, spirodiclofen, spirotetramat, spirotetramat enol, spirotetramat enol-glucoside, spirotetramat ketohydroxy, spirotetramat monohydroxy, spiroxamine, sulfosulfuron, sulfoxaflor, tebuconazole, tebufenozide, tebufenpyrad, teflubenzuron, terbufos, terbufos oxon, terbufos sulphone, terbufos sulphoxide, terbuthylazine, TFNA, TFNA-AM, TFNG, thiabendazole, thiacloprid, thiamethoxam, thiencarbazone-methyl, thifensulfuron-methyl, thiodicarb, thiometon, thiophanate-methyl, tolfenpyrad, topramezone, tralkoxydim, trichlorfon, tricyclazole, tridemorph, triflumizole, triflumuron, triticonazole, tritosulfuron, zoxamide	
	cyhexatin, dithianon, dodine, fenbutatin oxide, flonicamid, chlormequat chloride, mepiquat chloride	0.005
	fentin	0.0025

*- not accredited method

Supplementary Table S3. Methods details.

No.	Substance name	LOD (mg/kg)	LOQ (mg/kg)	Working Range (mg/kg)	Recoveries (%)	RSD (%)	Measurement Uncertainty (%)
**MULTI GC METHOD**							
1.	2-Phenylphenol	0,001	0,005	(0.005 - 5)	97	12	34,5
2.	Acetochlor	0,001	0,005	(0.005 - 5)	97	8,3	26,8
3.	Acrinathrin	0,001	0,005	(0.005 - 5)	90	9,95	35,7
4.	Alachlor	0,001	0,005	(0.005 - 5)	99	10	29,6
5.	Aldrin	0,0005	0,001	(0.001 - 5)	98	6,5	21,4
6.	Allethrin	0,001	0,005	(0.005 - 5)	108	8,8	31,5
7.	Ametryn	0,001	0,005	(0.005 - 5)	95	7,6	24,7
8.	Aminocarb	0,001	0,005	(0.005 - 5)	83	12	48,6
9.	Anthraquinone	0,001	0,005	(0.005 - 5)	98	9,8	33,0
10.	Atrazine	0,001	0,005	(0.005 - 5)	101	9,7	27,8
11.	Azaconazole	0,001	0,005	(0.005 - 5)	92	11	37,2
12.	Azinphos ethyl	0,001	0,005	(0.005 - 5)	92	5,5	26,7
13.	Azinphos-methyl	0,001	0,005	(0.005 - 5)	97	12	38,9
14.	Azoxystrobin	0,001	0,005	(0.005 - 5)	99	13	35,8
15.	Beflubutamid	0,001	0,005	(0.005 - 5)	88	5,5	28,1
16.	Benalaxyl	0,001	0,005	(0.005 - 5)	102	6,6	19,5
17.	Benfluralin	0,001	0,005	(0.005 - 5)	88	4,5	32,2
18.	Benfuracarb	0,001	0,005	(0.005 - 5)	93	6,7	32,8
19.	Bifenazate	0,001	0,005	(0.005 - 5)	93	9,7	36,7
20.	Bifenazate diazene	0,001	0,005	(0.005 - 5)	86	9,1	26,0
21.	Bifenox	0,001	0,005	(0.005 - 5)	110	15	46,4
22.	Bifenthrin	0,001	0,005	(0.005 - 5)	98	5,2	18,5
23.	Biphenyl	0,001	0,005	(0.005 - 5)	92	5,7	30,5
24.	Bitertanol	0,001	0,005	(0.005 - 5)	96	5,2	18,0
25.	Boscalid	0,001	0,005	(0.005 - 5)	103	6,4	22,0
26.	Bromfenvinphos	0,001	0,005	(0.005 - 5)	97	7,9	26,3
27.	Bromocyclen	0,001	0,005	(0.005 - 5)	98	7,8	24,4
28.	Bromophos	0,001	0,005	(0.005 - 5)	105	8,8	26,8
29.	Bromophos-ethyl	0,001	0,005	(0.005 - 5)	97	5,00	19,7
30.	Bromopropylate	0,001	0,005	(0.005 - 5)	101	7,5	21,4

31.	Bupirimate	0,001	0,005	(0.005 - 5)	100	5,2	17,7
32.	Buprofezin	0,001	0,005	(0.005 - 5)	102	10	29,9
33.	Butachlor	0,001	0,005	(0.005 - 5)	98	12	38,9
34.	Butafenacil	0,001	0,005	(0.005 - 5)	107	15	45,3
35.	Butylate	0,001	0,005	(0.005 - 5)	97	12	34,5
36.	Captafol	0,001	0,005	(0.005 - 5)	101	8,4	27,0
37.	Captan	0,001	0,005	(0.005 - 5)	101	9,8	31,4
38.	Carbaryl	0,001	0,005	(0.005 - 5)	91	14	43,3
39.	Carboxin	0,001	0,005	(0.005 - 5)	102	13	39,8
40.	Chinomethionat	0,001	0,005	(0.005 - 5)	91	9,9	37,0
41.	Chlorbenside	0,001	0,005	(0.005 - 5)	98	6,7	29,1
42.	Chlorbufam	0,001	0,005	(0.005 - 5)	101	13,3	38,5
43.	Chlordane, -cis	0,001	0,005	(0.005 - 5)	95	11	31,0
44.	Chlordane, -oxy	0,002	0,01	(0.01 - 5)	98	5,4	15,0
45.	Chlordane, -trans	0,002	0,01	(0.01 - 5)	97	11	29,0
46.	Chlorfenapyr	0,001	0,005	(0.005 - 5)	102	4,5	22,9
47.	Chlorfenson	0,001	0,005	(0.005 - 5)	99	5,7	17,0
48.	Chlorfenvinphos	0,001	0,005	(0.005 - 5)	99	9,5	29,9
49.	Chlormephos	0,001	0,005	(0.005 - 5)	95,5	12	34,9
50.	Chlorobenzilate	0,001	0,005	(0.005 - 5)	100	5	14,7
51.	Chloropropylate	0,001	0,005	(0.005 - 5)	99	5,4	19,8
52.	Chlorothalonil	0,001	0,005	(0.005 - 5)	103	8,7	27,5
53.	Chlorpropham	0,001	0,005	(0.005 - 5)	108	6,3	23,8
54.	Chlorpyrifos	0,001	0,005	(0.005 - 5)	98	8	23,8
55.	Chlorpyrifos-methyl	0,001	0,005	(0.005 - 5)	103	6,1	18,1
56.	Chlorthal-dimethyl	0,001	0,005	(0.005 - 5)	99	3,8	12,5
57.	Chlorthion	0,001	0,005	(0.005 - 5)	92	9,8	36,9
58.	Chlorthiophos	0,001	0,005	(0.005 - 5)	96	7,8	27,7
59.	Clodinafop propargyl	0,001	0,005	(0.005 - 5)	89	7,4	42,1
60.	Clomazone	0,001	0,005	(0.005 - 5)	104	8,7	25,9
61.	Coumaphos	0,001	0,005	(0.005 - 5)	99	11	43,6
62.	Crimidine	0,001	0,005	(0.005 - 5)	103	12	34,6
63.	Cyanazine	0,001	0,005	(0.005 - 5)	105	8,2	30,4
64.	Cyanophenphos	0,001	0,005	(0.005 - 5)	105	3,9	17,8
65.	Cyanophos	0,001	0,005	(0.005 - 5)	98	12,6	41,7
66.	Cycloate	0,001	0,005	(0.005 - 5)	105	9	32,7

67.	Cyfluthrin	0,001	0,005	(0.005 - 5)	99	9,9	29,1
68.	Cypermethrin	0,001	0,005	(0.005 - 5)	96	11	31,4
69.	Cyprazine	0,002	0,01	(0.01 - 5)	103	12	23,9
70.	Cyproconazole	0,001	0,005	(0.005 - 5)	96	7,2	39,4
71.	Cyprodinil	0,001	0,005	(0.005 - 5)	109	7,3	26,6
72.	DDD-o,p'	0,001	0,005	(0.005 - 5)	107	5,5	20,8
73.	DDD-p,p'	0,001	0,005	(0.005 - 5)	109	4,6	23,0
74.	DDE-o,p'	0,001	0,005	(0.005 - 5)	94	5,7	19,9
75.	DDE-p,p'	0,001	0,005	(0.005 - 5)	108	4,4	19,6
76.	DDM	0,001	0,005	(0.005 - 5)	99	3,8	12,5
77.	DDT-o,p'	0,001	0,005	(0.005 - 5)	88	8,2	33,7
78.	DDT-p,p'	0,001	0,005	(0.005 - 5)	92	6,1	24,0
79.	Deltamethrin	0,001	0,005	(0.005 - 5)	90	9,7	34,4
80.	Demeton-S	0,001	0,005	(0.005 - 5)	96	6,6	27,8
81.	Desmetryn	0,001	0,005	(0.005 - 5)	102	10,3	32,9
82.	Dialifos	0,001	0,005	(0.005 - 5)	99	5,5	21,7
83.	Diazinon	0,001	0,005	(0.005 - 5)	99	6	18,2
84.	Dichlobenil	0,001	0,005	(0.005 - 5)	108	4,8	20,3
85.	Dichlofenthion	0,001	0,005	(0.005 - 5)	109	10	33,7
86.	Dichlofluanid	0,001	0,005	(0.005 - 5)	88	9,7	36,5
87.	Dichloroaniline, 3,5-	0,001	0,005	(0.005 - 5)	99	16	47,9
88.	Dichlorobenzamide, 2,6-	0,002	0,01	(0.01 - 5)	90	11	22,9
89.	Dichlorobenzophenone-p,p	0,001	0,005	(0.005 - 5)	96	3,8	17,4
90.	Dichlorvos	0,001	0,005	(0.005 - 5)	98	6,7	21,8
91.	Diclobutrazol	0,001	0,005	(0.005 - 5)	92	11	34,0
92.	Dicloran	0,001	0,005	(0.005 - 5)	97	12	36,4
93.	Dicofol	0,001	0,005	(0.005 - 5)	102	6,4	20,7
94.	Dieldrin	0,0005	0,001	(0.001 - 5)	100	16	45,9
95.	Diethofencarb	0,001	0,005	(0.005 - 5)	99	10	36,2
96.	Difenoconazole	0,001	0,005	(0.005 - 5)	93	16	48,3
97.	Dimethachlor	0,001	0,005	(0.005 - 5)	100	5,3	17,8
98.	Dimethoate	0,001	0,005	(0.005 - 5)	97	7,5	27,2
99.	Dimethomorph	0,001	0,005	(0.005 - 5)	95	8,6	29,0
100.	Dimoxystrobin	0,001	0,005	(0.005 - 5)	97	4,2	33,2
101.	Diniconazole	0,001	0,005	(0.005 - 5)	91	8	33,5
102.	Dinitramine	0,002	0,01	(0.01 - 5)	83	5	48,6

103.	Dinobuton	0,002	0,01	(0.01 - 5)	92	14	44,4
104.	Dinoseb	0,002	0,01	(0.01 - 5)	90	7,9	22,0
105.	Dioxabenzofos	0,001	0,005	(0.005 - 5)	98	14	40,7
106.	Dioxacarb	0,001	0,005	(0.005 - 5)	86	6,8	34,0
107.	Dioxathion	0,001	0,005	(0.005 - 5)	96	12	37,4
108.	Diphenylamine	0,001	0,005	(0.005 - 5)	104	5	22,8
109.	Disulfoton	0,0005	0,001	(0.001 - 5)	98	7	26,4
110.	Disulfoton sulfon	0,002	0,01	(0.01 - 5)	88	5,4	15,0
111.	Disulfoton sulfoxide	0,002	0,01	(0.01 - 5)	75	11	30,0
112.	Ditalimfos	0,001	0,005	(0.005 - 5)	93	9,2	36,0
113.	DMST	0,001	0,005	(0.005 - 5)	104	8	24,0
114.	Dodemorph	0,001	0,005	(0.005 - 5)	93	9,3	34,4
115.	Edifenphos	0,001	0,005	(0.005 - 5)	104	9,7	28,2
116.	Endosulfan alpha	0,001	0,005	(0.005 - 5)	110	9,2	32,6
117.	Endosulfan beta	0,001	0,005	(0.005 - 5)	105	5,5	31,4
118.	Endosulfan sulphate	0,001	0,005	(0.005 - 5)	96	3,8	15,0
119.	Endrin	0,001	0,0025	(0.0025 - 5)	93	6,9	28,0
120.	Endrin keton	0,002	0,01	(0.01 - 5)	87	3,2	9,0
121.	EPN	0,001	0,005	(0.005 - 5)	109	5,2	22,8
122.	Epoxiconazole	0,001	0,005	(0.005 - 5)	97	5,2	33,1
123.	Esfenvalerate	0,001	0,005	(0.005 - 5)	94	10	39,9
124.	Etaconazole	0,001	0,005	(0.005 - 5)	99	8,1	26,8
125.	Ethalfluralin	0,001	0,005	(0.005 - 5)	87	5,2	35,0
126.	Ethion	0,001	0,005	(0.005 - 5)	97	3,3	27,0
127.	Ethofumesate	0,001	0,005	(0.005 - 5)	105	7,3	23,0
128.	Ethofumesate, -2-keto	0,002	0,01	(0.01 - 5)	80	4	11,0
129.	Ethoprophos	0,001	0,005	(0.005 - 5)	97	4,3	14,2
130.	Ethoxyquin	0,001	0,005	(0.005 - 5)	89	7,8	32,0
131.	Etofenprox	0,001	0,005	(0.005 - 5)	83	7,4	40,6
132.	Etrimfos	0,001	0,005	(0.005 - 5)	101	4,9	15,5
133.	Fenamiphos	0,001	0,005	(0.005 - 5)	103	11	33,1
134.	Fenarimol	0,001	0,005	(0.005 - 5)	109	2,2	19,7
135.	Fenazaquin	0,001	0,005	(0.005 - 5)	101	12	33,6
136.	Fenbuconazole	0,001	0,005	(0.005 - 5)	86	9,3	38,8
137.	Fenchlorphos	0,001	0,005	(0.005 - 5)	89	8,9	35,3
138.	Fenhexamid	0,001	0,005	(0.005 - 5)	103	7,1	21,3

139.	Fenitrothion	0,001	0,005	(0.005 - 5)	94	3,2	24,3
140.	Fenoxycarb	0,001	0,005	(0.005 - 5)	95	12	39,4
141.	Fenpropathrin	0,001	0,005	(0.005 - 5)	92	4,9	21,1
142.	Fenpropidin	0,001	0,005	(0.005 - 5)	103	5,7	23,1
143.	Fenpropimorph	0,001	0,005	(0.005 - 5)	88	5,7	29,2
144.	Fenpyrazamine	0,002	0,01	(0.01 - 5	83	9	25,0
145.	Fenthion	0,001	0,005	(0.005 - 5)	95	5,9	19,2
146.	Fenvalerate	0,001	0,005	(0.005 - 5)	94	10	39,9
147.	Fipronil	0,0005	0,001	(0.001 - 5)	106	6,6	22,1
148.	Fipronil desulfinyl	0,001	0,0025	(0.0025 - 5)	92	6,9	27,6
149.	Fipronil sulfon	0,001	0,0025	(0.0025 - 5)	99	13	25,3
150.	Fluchloralin	0,001	0,005	(0.005 - 5)	94	8,7	29,6
151.	Flucythrinate	0,001	0,005	(0.005 - 5)	89	7,6	31,1
152.	Fludioxonil	0,001	0,005	(0.005 - 5)	93	12	45,5
153.	Fluensulfone	0,002	0,01	(0.01 - 5)	81	4,3	12,0
154.	Flumetralin	0,001	0,005	(0.005 - 5)	87	5,9	41,9
155.	Flumioxazin	0,002	0,01	(0.01 - 5)	92	4	13,0
156.	Fluorodifen	0,001	0,005	(0.005 - 5)	97	11	33,7
157.	Fluotrimazole	0,001	0,005	(0.005 - 5)	99	7,7	25,6
158.	Fluquinconazole	0,001	0,005	(0.005 - 5)	91	6,8	26,1
159.	Flurprimidol	0,002	0,01	(0.01 - 5)	89	3,1	8,6
160.	Flurtamone	0,002	0,01	(0.01 - 5)	83	18	36,1
161.	Flusilazole	0,001	0,005	(0.005 - 5)	98	5	21,7
162.	Flutianil	0,002	0,01	(0.01 - 5)	77	3,6	10,0
163.	Flutriafol	0,001	0,005	(0.005 - 5)	98	8	25,0
164.	Folpet	0,001	0,005	(0.005 - 5)	92	13	41,5
165.	Fonofos	0,001	0,005	(0.005 - 5)	107	7,6	25,4
166.	Formothion	0,001	0,005	(0.005 - 5)	102	7,4	21,6
167.	Furalaxyl	0,001	0,005	(0.005 - 5)	104	6,7	20,5
168.	Furathiocarb	0,001	0,005	(0.005 - 5)	104	13	42,7
169.	Gamma-cyhalothrin	0,001	0,005	(0.005 - 5)	107	13	42,9
170.	Halfenprox	0,001	0,005	(0.005 - 5)	90	7,0	45,0
171.	Heptachlor	0,0005	0,001	(0.001 - 5)	87	6,5	31,5
172.	Heptachlor cis-epoxid	0,001	0,0025	(0.0025 - 5)	97	6,3	30,0
173.	Heptachlor trans-epoxid	0,001	0,0025	(0.0025 - 5)	103	9	17,9
174.	Heptenophos	0,001	0,005	(0.005 - 5)	102	9,1	28,0

175.	Hexachlorobenzene	0,0005	0,001	(0.001 - 5)	107	6,2	25,1
176.	Hexachlorocyclohexane (HCH), alpha	0,001	0,005	(0.005 - 5)	106	5,3	19,7
177.	Hexachlorocyclohexane (HCH), beta	0,001	0,005	(0.005 - 5)	105	7,8	24,3
178.	Hexaconazole	0,001	0,005	(0.005 - 5)	99	9,3	31,5
179.	Imazalil	0,001	0,005	(0.005 - 5)	101	13	39,3
180.	Iodofenphos	0,001	0,005	(0.005 - 5)	97	8,2	25,4
181.	Iprobenfos	0,001	0,005	(0.005 - 5)	101	11	31,9
182.	Iprodione	0,001	0,005	(0.005 - 5)	94	6,8	24,9
183.	Isocarbophos	0,001	0,005	(0.005 - 5)	109	8,8	30,3
184.	Isofenphos	0,001	0,005	(0.005 - 5)	104	6,7	21,0
185.	Isofenphos-methyl	0,001	0,005	(0.005 - 5)	96	6,5	21,1
186.	Isofetamid	0,002	0,01	(0.01 - 5)	76	2,7	7,7
187.	Kresoxim-methyl	0,001	0,005	(0.005 - 5)	104	8,5	25,1
188.	lambda-Cyhalothrin	0,001	0,005	(0.005 - 5)	99	8,4	26,6
189.	Lindane	0,001	0,005	(0.005 - 5)	102	8,1	25,9
190.	Malaoxon	0,001	0,005	(0.005 - 5)	96	6,7	21,6
191.	Malathion	0,001	0,005	(0.005 - 5)	109	5,4	23,4
192.	Mandestrobin	0,002	0,01	(0.01 - 5)	77	4,2	12,0
193.	Mecarbam	0,001	0,005	(0.005 - 5)	103	7,6	23,7
194.	Mepanipyrim	0,001	0,005	(0.005 - 5)	94	10	31,9
195.	Mepronil	0,001	0,005	(0.005 - 5)	97	9,3	31,0
196.	Metalaxyl	0,001	0,005	(0.005 - 5)	100	12	36,5
197.	Metazachlor	0,001	0,005	(0.005 - 5)	107	7,1	24,8
198.	Metconazole	0,001	0,005	(0.005 - 5)	106	8	25,8
199.	Methacrifos	0,001	0,005	(0.005 - 5)	110	6,4	26,4
200.	Methidathion	0,001	0,005	(0.005 - 5)	106	5,1	19,3
201.	Methoxychlor	0,001	0,005	(0.005 - 5)	101	11	32,8
202.	Metolachlor	0,001	0,005	(0.005 - 5)	108	4,4	20,5
203.	Metribuzin	0,001	0,005	(0.005 - 5)	110	5,5	25,0
204.	Mevinphos	0,001	0,005	(0.005 - 5)	92	11	34,9
205.	Molinate	0,002	0,01	(0.01 - 5)	94	6,2	17,0
206.	Myclobutanil	0,001	0,005	(0.005 - 5)	99	6,7	23,8
207.	Nitralin	0,001	0,005	(0.005 - 5)	87	5,7	40,3
208.	Nitrapyrin	0,001	0,005	(0.005 - 5)	92	8	27,8
209.	Nitrofen	0,0005	0,001	(0.001 - 5)	87	9,1	36,1
210.	Nitrothal isopropyl	0,001	0,005	(0.005 - 5)	90	7,3	39,2

211.	Nuarimol	0,001	0,005	(0.005 - 5)	92	10	30,2
212.	Oxadiazon	0,002	0,01	(0.01 - 5)	92	3,2	9,0
213.	Oxadixyl	0,001	0,005	(0.005 - 5)	106	4,7	18,2
214.	Oxyfluorfen	0,001	0,005	(0.005 - 5)	101	8	24,9
215.	Paclobutrazol	0,001	0,005	(0.005 - 5)	85	6,7	35,8
216.	Parathion	0,001	0,005	(0.005 - 5)	101	11	30,3
217.	Parathion-methyl	0,001	0,005	(0.005 - 5)	101	6,5	20,6
218.	Penconazole	0,001	0,005	(0.005 - 5)	104	6,2	19,3
219.	Pencycuron	0,001	0,005	(0.005 - 5)	99	7,7	24,1
220.	Pendimethalin	0,001	0,005	(0.005 - 5)	103	11	32,2
221.	Penflufen	0,002	0,01	(0.01 - 5)	91	4	11,0
222.	Pentachloroaniline	0,002	0,01	(0.01 - 5)	96	11	31,0
223.	Permethrin	0,001	0,005	(0.005 - 5)	89	8,5	32,9
224.	Perthane (Ethylan)	0,001	0,005	(0.005 - 5)	100	3,6	15,7
225.	Pethoxamid	0,001	0,005	(0.005 - 5)	89	3	10,0
226.	Phenthoate	0,001	0,005	(0.005 - 5)	97	3,8	21,1
227.	Phorate	0,001	0,005	(0.005 - 5)	103	6,3	22,2
228.	Phorate sulfone	0,002	0,01	(0.01 - 5)	90	16	32,8
229.	Phorate sulfoxide	0,001	0,005	(0.005 - 5)	102	7,7	23,2
230.	Phosalone	0,001	0,005	(0.005 - 5)	89	6,8	29,0
231.	Phosmet	0,001	0,005	(0.005 - 5)	84	4,6	34,8
232.	Phosphamidon	0,001	0,005	(0.005 - 5)	91	10	34,0
233.	Phthalimide	0,001	0,005	(0.005 - 5)	100	7,8	22,7
234.	Picolinafen	0,001	0,005	(0.005 - 5)	106	8,9	32,8
235.	Picoxystrobin	0,001	0,005	(0.005 - 5)	107	9,7	30,7
236.	Piperonyl butoxide	0,001	0,005	(0.005 - 5)	101	14	41,0
237.	Piperophos	0,001	0,005	(0.005 - 5)	94	7,9	33,7
238.	Pirimicarb	0,001	0,005	(0.005 - 5)	105	5,7	19,1
239.	Pirimicarb desmethyl	0,001	0,005	(0.005 - 5)	90	7,2	29,2
240.	Pirimiphos-ethyl	0,002	0,01	(0.01 - 5)	83	4	10,0
241.	Pirimiphos-methyl	0,001	0,005	(0.005 - 5)	107	6,4	23,2
242.	Procymidone	0,001	0,005	(0.005 - 5)	101	4,7	16,9
243.	Profenofos	0,001	0,005	(0.005 - 5)	86	6,5	32,7
244.	Profluralin	0,001	0,005	(0.005 - 5)	101	6,2	20,3
245.	Prometon	0,001	0,005	(0.005 - 5)	102	11	34,6
246.	Prometryn	0,001	0,005	(0.005 - 5)	103	6,3	19,4

247.	Propachlor	0,001	0,005	(0.005 - 5)	96	9,5	29,9
248.	Propargite	0,001	0,005	(0.005 - 5)	92	5,5	22,8
249.	Propazine	0,001	0,005	(0.005 - 5)	101	5,3	15,8
250.	Propetamphos	0,001	0,005	(0.005 - 5)	98	10	30,6
251.	Propham	0,001	0,005	(0.005 - 5)	106	6	20,7
252.	Propiconazole	0,001	0,005	(0.005 - 5)	96	12	36,3
253.	Propyzamide	0,001	0,005	(0.005 - 5)	101	3,5	15,6
254.	Prothioconazole destio	0,001	0,005	(0.005 - 5)	94	6,6	22,1
255.	Prothiofos	0,001	0,005	(0.005 - 5)	94	9,2	28,9
256.	Pyraclostrobin	0,001	0,005	(0.005 - 5)	92	12	37,7
257.	Pyrazophos	0,001	0,005	(0.005 - 5)	91	4,4	31,2
258.	Pyrethrins	0,01	0,05	(0.05 - 5)	87	7,1	34,0
259.	Pyridaben	0,001	0,005	(0.005 - 5)	103	6,1	22,3
260.	Pyridalyl	0,002	0,01	(0.01 - 5)	90	8	23,0
261.	Pyridaphenthion	0,002	0,01	(0.01 - 5)	82	7,4	21,0
262.	Pyrifenox	0,001	0,005	(0.005 - 5)	95	8,3	30,8
263.	Pyrimethanil	0,001	0,005	(0.005 - 5)	101	3,6	14,6
264.	Pyriofenone	0,002	0,01	(0.01 - 5)	76	4	11,0
265.	Pyriproxyfen	0,001	0,005	(0.005 - 5)	92	9	30,7
266.	Pyroquilon	0,001	0,005	(0.005 - 5)	97	12	40,5
267.	Quinalphos	0,001	0,005	(0.005 - 5)	105	11	34,9
268.	Quinoxyfen	0,001	0,005	(0.005 - 5)	104	5,9	18,4
269.	Quintozene	0,001	0,005	(0.005 - 5)	105	11	36,1
270.	Resmethrin	0,001	0,005	(0.005 - 5)	91	7,6	29,9
271.	Silafluofen	0,002	0,01	(0.01 - 5)	88	3,5	9,8
272.	Simazine	0,002	0,01	(0.01 - 5)	100	11	33,0
273.	Spiromesifen	0,001	0,005	(0.005 - 5)	101	8,3	24,7
274.	Sulfotep	0,001	0,005	(0.005 - 5)	101	10	30,2
275.	tau-Fluvalinate	0,001	0,005	(0.005 - 5)	95	11	35,5
276.	Tebuconazole	0,001	0,005	(0.005 - 5)	90	11	36,6
277.	Tebufenpyrad	0,001	0,005	(0.005 - 5)	105	4,4	29,0
278.	Tecnazene	0,001	0,005	(0.005 - 5)	104	6,7	20,5
279.	Tefluthrin	0,001	0,005	(0.005 - 5)	101	2,9	12,5
280.	Terbacil	0,001	0,005	(0.005 - 5)	94	5,2	21,4
281.	Terbufos	0,0005	0,001	(0.001 - 5)	104	3,7	15,3
282.	Terbutryn	0,001	0,005	(0.005 - 5)	98	2,8	12,5

283.	Tetrachlorvinphos	0,001	0,005	(0.005 - 5)	88	12	40,7
284.	Tetraconazole	0,001	0,005	(0.005 - 5)	90	7,6	28,9
285.	Tetradifon	0,001	0,005	(0.005 - 5)	97	5	17,9
286.	Tetrahydrophthalimide	0,001	0,005	(0.005 - 5)	102	13	39,7
287.	Tetramethrin	0,001	0,005	(0.005 - 5)	90	5,2	16,5
288.	Tetrasul	0,001	0,005	(0.005 - 5)	96	5,6	18,1
289.	Thiobencarb	0,002	0,01	(0.01 - 5)	95	9,4	18,8
290.	Tolclofos-methyl	0,001	0,005	(0.005 - 5)	101	8,2	23,3
291.	Tolylfluanid	0,001	0,005	(0.005 - 5)	85	7,9	37,9
292.	Triadimefon	0,001	0,005	(0.005 - 5)	97	5,3	26,9
293.	Triadimenol	0,001	0,005	(0.005 - 5)	84	7,6	39,1
294.	Tri-allate	0,001	0,005	(0.005 - 5)	102	5,8	19,0
295.	Triazophos	0,001	0,005	(0.005 - 5)	100	9,8	29,5
296.	Trifloxystrobin	0,001	0,005	(0.005 - 5)	95	4,7	18,6
297.	Triflumizole	0,001	0,005	(0.005 - 5)	92	5,8	26,5
298.	Trifluralin	0,001	0,005	(0.005 - 5)	104	4,4	15,6
299.	Vinclozolin	0,001	0,005	(0.005 - 5)	103	4,5	16,8
**MULTI LC METHOD**							
1.	Avermectin	0,002	0,01	(0.01-2)	93	14,0	44,0
2.	Acephate	0,002	0,01	(0.01-2)	86	7,9	35,8
3.	Acetamiprid	0,001	0,005	(0.005-2)	91	3,0	19,1
4.	Aclonifen	0,0025	0,01	(0.01-1)	104	8,7	34,9
5.	Aldicarb	0,002	0,01	(0.01-2)	97	3,5	11,7
6.	Aldicarb sulfone	0,005	0,01	(0.01-2)	105	3,2	19,3
7.	Aldicarb sulfoxide	0,001	0,01	(0.01-2)	95	2,5	13,1
8.	Ametoctradin	0,001	0,005	(0.005-2)	91	5,5	24,0
9.	Amidosulfuron	0,001	0,005	(0.005-2)	80	3,5	41,3
10.	Amisulbrom	0,0025	0,01	(0.01-1)	100	6,2	21,8
11.	Azadirachtin	0,002	0,01	(0.01-2)	82	13,0	46,0
12.	Aziprotryne	0,0025	0,01	(0.01-1)	102	3,8	17,5
13.	Azoxystrobin	0,001	0,005	(0.005-2)	91	4,7	27,1
14.	BAC C10	0,002	0,01	(0.01-2)	101	5,0	12,0
15.	BAC C12	0,002	0,01	(0.01-2)	99	5,0	15,0
16.	BAC C14	0,002	0,01	(0.01-2)	82	5,0	41,0
17.	BAC C8	0,002	0,01	(0.01-2)	95	3,0	14,0

18.	Beflubutamid	0,0025	0,01	(0.01-1)	103	3,7	16,7
19.	Bendiocarb	0,0025	0,01	(0.01-1)	103	5,3	19,3
20.	Benthiavalicarb isopropyl	0,0025	0,01	(0.01-1)	102	2,5	20,8
21.	Benzovindiflupyr	0,001	0,005	(0.005-2)	110	7,0	27,0
22.	Bixafen	0,0025	0,01	(0.01-1)	105	4,8	29,3
23.	Boscalid	0,002	0,005	(0.005-2)	89	5,2	27,1
24.	Bromacil	0,0025	0,01	(0.01-1)	102	2,5	14,4
25.	Bromuconazole	0,004	0,01	(0.01-2)	97	7,1	21,0
26.	Cadusafos	0,001	0,0025	(0.0025-0.5)	94	3,5	16,2
27.	Carbaryl	0,0005	0,005	(0.005-2)	97	4,9	15,4
28.	Carbendazim	0,001	0,005	(0.005-2)	94	3,8	16,3
29.	Carbetamide	0,0025	0,01	(0.01-1)	101	2,5	15,1
30.	Carbofuran	0,0005	0,005	(0.005-2)	98	2,9	14,9
31.	Carbofuran 3-hydroxy	0,001	0,005	(0.005-2)	103	6,7	19,8
32.	Carbofuran 3-keto	0,001	0,01	(0.01-2)	98	3,0	17,0
33.	Carfentrazone-ethyl	0,002	0,01	(0.01-2)	102	10,0	21,0
34.	Chlorantraniliprole	0,001	0,005	(0.005-2)	96	5,3	18,7
35.	Chloridazon	0,001	0,005	(0.005-2)	97	3,2	15,5
36.	Chlorotoluron	0,001	0,005	(0.005-2)	100	3,7	11,1
37.	Chlorpyrifos	0,0025	0,01	(0.01-1)	103	3,0	23,3
38.	Chlorsulfuron	0,002	0,005	(0.005-2)	76	2,9	48,5
39.	Chromafenozide	0,0025	0,01	(0.01-1)	103	2,2	16,2
40.	Clodinafop propargyl	0,002	0,01	(0.01-2)	96	3,9	16,0
41.	Clofentezine	0,0005	0,005	(0.005-2)	95	3,7	14,1
42.	Clothianidin	0,002	0,01	(0.01-2)	103	4,3	16,4
43.	Cyantraniliprole	0,002	0,01	(0.01-2)	84	16,0	36,0
44.	Cyazofamid	0,001	0,005	(0.005-2)	97	3,3	11,0
45.	Cycloxydim	0,001	0,005	(0.005-2)	96	3,2	11,9
46.	Cyflufenamid	0,001	0,005	(0.005-2)	93	3,6	17,9
47.	Cyflumetofen	0,001	0,005	(0.005-2)	108	7,0	19,0
48.	Cymiazol	0,0025	0,01	(0.01-1)	100	2,9	21,6
49.	Cymoxanil	0,001	0,005	(0.005-2)	98	3,0	9,2
50.	Cyproconazole	0,002	0,01	(0.01-2)	101	3,0	13,3
51.	DDAC C8	0,002	0,01	(0.01-2)	102	5,0	15,0
52.	DEET	0,002	0,01	(0.01-2)	100	7,9	22,7
53.	Demeton-S-methyl	0,001	0,0025	(0.0025-0.5)	93	9,7	17,1

54.	Demeton-S-methyl-sulphone	0,001	0,0025	(0.0025-0.5)	94	3,9	15,8
55.	Demeton-S-methyl-sulphoxide	0,001	0,0025	(0.0025-0.5)	92	7,0	25,5
56.	Desmedipham	0,001	0,01	(0.01-2)	97	3,0	10,6
57.	Dicrotophos	0,0025	0,01	(0.01-1)	100	2,6	13,4
58.	Diethofencarb	0,0005	0,005	(0.005-2)	101	2,0	8,4
59.	Diflubenzuron	0,002	0,005	(0.005-2)	94	7,5	30,2
60.	Diflufenican	0,0025	0,01	(0.01-1)	101	3,3	26,0
61.	Dimethenamid	0,001	0,005	(0.005-2)	100	8,0	17,0
62.	Dimethoate	0,001	0,005	(0.005-2)	97	5,5	17,6
63.	Dinoterfuran	0,002	0,01	(0.01-2)	103	6,0	13,0
64.	Disulfoton	0,002	0,01	(0.01-2)	82	4,1	47,0
65.	Disulfoton sulfon	0,001	0,005	(0.005-2)	96	4,0	14,2
66.	Disulfoton sulfoxide	0,001	0,005	(0.005-2)	96	4,0	15,0
67.	Diuron	0,0025	0,01	(0.01-1)	102	3,1	12,4
68.	DMF	0,0005	0,005	(0.005-2)	100	6,3	18,2
69.	DMPF	0,0005	0,005	(0.005-2)	92	2,9	18,9
70.	Emamectin	0,0025	0,01	(0.01-1)	103	2,8	22,5
71.	Ethametsulfuron-methyl	0,001	0,005	(0.005-2)	96	8,0	20,0
72.	Ethiofencarb	0,0025	0,01	(0.01-1)	101	3,0	12,4
73.	Ethirimol	0,0025	0,01	(0.01-1)	94	2,9	18,6
74.	Etoxazole	0,0005	0,005	(0.005-2)	88	3,6	25,4
75.	Famoxadone	0,0025	0,01	(0.01-1)	100	4,1	30,9
76.	Fenamidone	0,001	0,005	(0.005-2)	94	3,2	15,6
77.	Fenamiphos	0,001	0,005	(0.005-2)	94	3,3	15,9
78.	Fenamiphos sulfoxide	0,0005	0,005	(0.005-2)	96	2,0	10,6
79.	Fenamiphos sulphone	0,001	0,005	(0.005-2)	99	3,0	10,4
80.	Fenbuconazole	0,002	0,005	(0.005-2)	102	10,3	33,7
81.	Fenfuram	0,0025	0,01	(0.01-1)	104	3,0	16,5
82.	Fenhexamid	0,0025	0,01	(0.01-1)	94	5,3	18,9
83.	Fenobucarb	0,0025	0,01	(0.01-1)	102	4,1	15,1
84.	Fenoxaprop-P-ethyl	0,0005	0,005	(0.005-2)	96	2,6	12,8
85.	Fenpropidin	0,0025	0,01	(0.01-1)	102	2,7	16,5
86.	Fenpropimorph	0,001	0,005	(0.005-2)	98	3,8	12,0
87.	Fenpyroximate	0,0005	0,005	(0.005-2)	99	3,7	15,7
88.	Fensulfothion	0,001	0,0025	(0.0025-0.5)	92	4,7	20,3
89.	Fensulfothion oxon	0,001	0,0025	(0.0025-0.5)	92	4	19,6

90.	Fensulfothion oxon sulphone	0,001	0,0025	(0.0025-0.5)	99	5,7	16,4
91.	Fensulfothion sulphone	0,001	0,0025	(0.0025-0.5)	94	4,8	18,7
92.	Fenthion	0,0025	0,01	(0.01-1)	103	9,7	39,5
93.	Fenthion oxon	0,002	0,01	(0.01-2)	92	2,8	17
94.	Fenthion oxon sulphone	0,002	0,01	(0.01-2)	88	2,4	25
95.	Fenthion sulfoxide	0,0025	0,01	(0.01-1)	105	3,2	14,3
96.	Fenthion sulphone	0,002	0,01	(0.01-2)	91	3,5	20,0
97.	Flazasulfuron	0,001	0,005	(0.005-2)	96	8,0	20,0
98.	Flonicamid	0,001	0,005	(0.005-2)	94	2,7	15,1
99.	Florasulam	0,002	0,01	(0.01-2)	84	5,0	43,0
100.	Flufenacet	0,001	0,005	(0.005-2)	99	3,4	10,6
101.	Flufenoxuron	0,001	0,005	(0.005-2)	92	5,9	29,6
102.	Fluopicolide	0,001	0,005	(0.005-2)	99	8,0	16,0
103.	Fluopyram	0,001	0,005	(0.005-2)	101	4,0	11,0
104.	Fluoxastrobin	0,001	0,005	(0.005-2)	100	7,0	22,5
105.	Flupyradifurone	0,002	0,01	(0.01-2)	81	19,0	39,0
106.	Flurochloridone	0,005	0,01	(0.01-2)	96	9,2	27,6
107.	Flutianil	0,002	0,01	(0.01-2)	90	4,6	24,0
108.	Flutolanil	0,001	0,005	(0.005-2)	98	2,0	6,6
109.	Flutriafol	0,001	0,01	(0.01-2)	99	3,0	9,4
110.	Fluxapyroxad	0,0025	0,01	(0.01-1)	102	5,5	19,3
111.	Foramsulfuron	0,001	0,005	(0.005-2)	82	12,8	46,0
112.	Formetanate	0,001	0,01	(0.01-2)	101	4,2	16,9
113.	Fosthiazate	0,0025	0,01	(0.01-1)	102	2,9	10,0
114.	Fuberidazole	0,001	0,005	(0.005-2)	95	2,7	15,0
115.	Halofenozide	0,002	0,01	(0.01-2)	96	2,4	20,0
116.	Hexaflumuron	0,001	0,005	(0.005-2)	102	9,0	21,0
117.	Hexytiazox	0,001	0,005	(0.005-2)	97	3,8	12,5
118.	Imazalil	0,001	0,01	(0.01-2)	98	1,7	8,4
119.	Imazapic	0,002	0,01	(0.01-2)	101	5,0	16,0
120.	Imidacloprid	0,001	0,01	(0.01-2)	99	3,2	10,0
121.	Indoxacarb	0,001	0,005	(0.005-2)	100	5,4	16,3
122.	Iodosulfuron methyl	0,0025	0,01	(0.01-1)	84	4,5	34,6
123.	Ipconazole	0,0025	0,01	(0.01-1)	98	5,4	21,0
124.	Iprovalicarb	0,001	0,005	(0.005-2)	94	3,4	16,0
125.	Isofetamid	0,002	0,01	(0.01-2)	100	2,3	17,0

126.	Isoprocarb	0,0025	0,01	(0.01-1)	103	3,0	20,0
127.	Isoprothiolane	0,0025	0,01	(0.01-1)	101	9,3	18,0
128.	Isoproturon	0,001	0,005	(0.005-2)	99	2,5	10,2
129.	Isopyrazam	0,001	0,005	(0.005-2)	98	1,7	13,9
130.	Isoxaben	0,001	0,005	(0.005-2)	110	5,0	26,0
131.	Isoxaflutole	0,001	0,005	(0.005-2)	97	14,0	29,0
132.	Isoxathion	0,001	0,005	(0.005-2)	110	6,0	26,0
133.	Lenacil	0,001	0,01	(0.01-2)	93	5,2	20,4
134.	Linuron	0,001	0,005	(0.005-2)	98	8,0	24,4
135.	Lufenuron	0,002	0,01	(0.01-2)	86	11,0	44,0
136.	Malaoxon	0,001	0,005	(0.005-2)	96	2,1	15,1
137.	Malathion	0,001	0,01	(0.01-2)	102	4,2	13,6
138.	Mandipropamid	0,001	0,005	(0.005-2)	93	2,7	16,1
139.	Metaflumizone	0,002	0,01	(0.01-2)	100	9,0	26,0
140.	Metalaxyl	0,0005	0,005	(0.005-2)	98	2,8	10,8
141.	Metamitron	0,002	0,01	(0.01-2)	94	2,7	13,7
142.	Metazachlor	0,002	0,01	(0.01-2)	94	2,3	16,0
143.	Methamidophos	0,002	0,01	(0.01-2)	93	2,6	15,7
144.	Methiocarb	0,0005	0,005	(0.005-2)	102	3,4	10,9
145.	Methiocarb sulphone	0,001	0,01	(0.01-2)	92	6,3	23,8
146.	Methiocarb sulphoxide	0,0005	0,005	(0.005-2)	93	1,8	15,4
147.	Methomyl	0,001	0,01	(0.01-2)	99	3,6	10,6
148.	Methoprotryne	0,002	0,01	(0.01-2)	112	5,0	26,0
149.	Methoxyfenozide	0,002	0,005	(0.005-2)	100	2,2	9,3
150.	Metobromuron	0,002	0,01	(0.01-2)	100	5,0	13,0
151.	Metolachlor-S	0,001	0,005	(0.005-2)	93	4,9	19,5
152.	Metosulam	0,0025	0,01	(0.01-2)	92	13,7	44
153.	Metoxuron	0,0025	0,01	(0.01-1)	101	2,4	11,4
154.	Metrafenone	0,001	0,005	(0.005-2)	99	3,2	10,1
155.	Metsulfuron-methyl	0,002	0,005	(0.005-2)	79	4,9	44,9
156.	Monocrotophos	0,001	0,005	(0.005-2)	100	3,7	13,3
157.	Monuron	0,0025	0,01	(0.01-1)	105	4,9	18,1
158.	Napropamide	0,001	0,005	(0.005-2)	98	4,1	13,8
159.	Nicosulfuron	0,001	0,005	(0.005-2)	88	11,0	32,0
160.	Nicotine	0,0025	0,01	(0.01-2)	93	8,2	26,9
161.	Nitenpyram	0,002	0,01	(0.01-2)	106	8,0	18,0

162.	Novaluron	0,001	0,005	(0.005-2)	99	14,0	35,0
163.	Omethoate	0,001	0,0025	(0.0025-2)	90	7,4	28,6
164.	Oxadixyl	0,001	0,005	(0.005-2)	97	3,2	12,5
165.	Oxamyl	0,001	0,005	(0.005-2)	93	1,8	14,6
166.	Oxycarboxin	0,0025	0,01	(0.01-1)	100	2,8	12,6
167.	Paraoxon-methyl	0,001	0,005	(0.005-2)	101	3,5	10,2
168.	Parathion	0,001	0,01	(0.01-2)	100	8,6	27,5
169.	Parathion-methyl	0,0025	0,01	(0.01-1)	100	11,7	33,7
170.	Pencycuron	0,001	0,005	(0.005-2)	96	5,7	19,7
171.	Pendimethalin	0,002	0,005	(0.005-2)	102	2,0	13,2
172.	Penflufen	0,002	0,01	(0.01-2)	117	6,0	35,0
173.	Penthiopyrad	0,0025	0,01	(0.01-1)	103	3,7	14,3
174.	Pethoxamid	0,0025	0,01	(0.01-0.5)	94	6,1	20,5
175.	Phenmedipham	0,001	0,01	(0.01-2)	97	4,4	14,1
176.	Phenthoate	0,001	0,005	(0.005-2)	96	3,0	12,0
177.	Phosmet	0,001	0,005	(0.005-2)	97	2,8	10,0
178.	Phosmet okson	0,002	0,01	(0.01-2)	109	7,7	27,6
179.	Phoxim	0,002	0,01	(0.01-2)	104	8,0	22,0
180.	Pinoxaden	0,0005	0,005	(0.005-2)	92	3,7	19,6
181.	Piperonyl butoxide	0,0025	0,01	(0.01-1)	103	2,5	16,3
182.	Prochloraz	0,001	0,005	(0.005-2)	98	2,7	8,5
183.	Prochloraz BTS 44595	0,0025	0,01	(0.01-1)	104	3,0	21,6
184.	Prochloraz BTS 44596	0,0025	0,01	(0.01-1)	101	3,4	14,8
185.	Propamocarb	0,001	0,005	(0.005-2)	80	3,8	42,1
186.	Propaquizafop	0,001	0,005	(0.005-2)	98	3,1	11,5
187.	Propoxur	0,0025	0,01	(0.01-1)	101	2,6	14,9
188.	Propoxycarbazone	0,0025	0,01	(0.01-1)	85	4,6	33,0
189.	Proquinazid	0,001	0,005	(0.005-2)	97	1,3	10,2
190.	Prosulfocarb	0,001	0,005	(0.005-2)	96	2,8	15,7
191.	Prosulfuron	0,001	0,005	(0.005-2)	94	19,0	36,0
192.	Pymetrozine	0,005	0,02	(0.02-2)	79	2,8	49
193.	Pyridaben	0,001	0,005	(0.005-2)	93	2,7	15,8
194.	Pyridafol	0,002	0,01	(0.01-2)	79	3,9	47
195.	Pyrifenox	0,002	0,01	(0.01-2)	92	8,0	27,0
196.	Pyriproxyfen	0,0025	0,01	(0.01-1)	104	2,7	16,7
197.	Pyroquilon	0,0025	0,01	(0.01-1)	101	2,3	11,5

198.	Pyroxsulam	0,001	0,005	(0.005-2)	94	7,0	18,0
199.	Quinclorac	0,002	0,01	(0.01-2)	114	17,0	38,0
200.	Quinoclanmine	0,0025	0,01	(0.01-1)	105	5,2	26,8
201.	Quizalofop-ethyl	0,001	0,005	(0.005-2)	96	2,2	11,8
202.	Rimsulfuron	0,001	0,01	(0.01-2)	84	4,2	44,7
203.	Rotenone	0,0025	0,01	(0.01-1)	103	3,5	27,0
204.	Saflufenacil	0,001	0,005	(0.005-2)	102	12,0	26,0
205.	Silthiofam	0,001	0,005	(0.005-2)	95	6,2	20,4
206.	Spinetoram	0,0025	0,01	(0.01-1)	102	2,6	22,0
207.	Spinosyn A	0,001	0,005	(0.005-2)	94	2,5	13,5
208.	Spinosyn D	0,001	0,005	(0.005-2)	95	1,9	13,7
209.	Spirodiclofen	0,001	0,005	(0.005-2)	92	2,5	17,7
210.	Spirotetramat	0,001	0,005	(0.005-2)	95	3,5	15,8
211.	Spirotetramat enol	0,001	0,005	(0.005-2)	78	3,9	48,1
212.	Spirotetramat enol-glucoside	0,002	0,005	(0.005-2)	85	3,9	42,0
213.	Spirotetramat ketohydroxy	0,001	0,005	(0.005-2)	92	9,4	18,7
214.	Spirotetramat monohydroxy	0,001	0,005	(0.005-2)	94	5,4	19,4
215.	Spiroxamine	0,001	0,005	(0.005-2)	94	2,7	14,4
216.	Sulfometuron methyl	0,002	0,005	(0.005-2)	77	3,5	47,4
217.	Sulfosulfuron	0,0025	0,01	(0.01-1)	90	4,4	25,8
218.	Sulfoxaflor	0,002	0,01	(0.01-2)	97	12,0	10,0
219.	Tebuconazole	0,002	0,01	(0.01-2)	97	7,3	24,4
220.	Tebufenozide	0,001	0,005	(0.005-2)	93	2,5	17,1
221.	Tebufenpyrad	0,001	0,005	(0.005-2)	99	5,4	17,0
222.	Teflubenzuron	0,005	0,01	(0.01-2)	93	6,7	23,7
223.	Tepraloxydim	0,005	0,01	(0.01-2)	97	6,5	19,6
224.	Terbufos	0,0025	0,01	(0.01-0.5)	94	6,1	21,0
225.	Terbufos oxon	0,002	0,01	(0.01-2)	85	3,0	32,0
226.	Terbufos sulphone	0,0025	0,01	(0.01-0.5)	93	5,9	21,9
227.	Terbufos sulphoxide	0,001	0,005	(0.005-2)	93	7,8	26,7
228.	Terbuthylazine	0,001	0,005	(0.005-2)	97	2,2	9,4
229.	Thiabendazole	0,001	0,005	(0.005-2)	93	1,7	15,5
230.	Thiacloprid	0,001	0,005	(0.005-2)	98	2,0	9,7
231.	Thiamethoxam	0,001	0,005	(0.005-2)	97	2,2	12,5
232.	Thiencarbazone-methyl	0,001	0,005	(0.005-2)	97	14,0	33,0
233.	Thifensulfuron-methyl	0,002	0,01	(0.01-2)	76	4,0	49,0

234.	Thiodicarb	0,0005	0,005	(0.005-2)	93	2,7	15,7
235.	Thiometon	0,0025	0,01	(0.01-1)	96	7,2	22,0
236.	Thiophanate-methyl	0,001	0,005	(0.005-2)	97	1,9	10,6
237.	Tolfenpyrad	0,002	0,01	(0.01-2)	80	2,0	39,0
238.	Topramezone	0,002	0,01	(0.01-2)	98	5,0	11,0
239.	Tralkoxydim	0,0025	0,01	(0.01-1)	92	3,3	22,0
240.	Trichlorfon	0,002	0,01	(0.01-2)	96	9,0	24,0
241.	Tricyclazole	0,0025	0,01	(0.01-1)	97	2,4	15,8
242.	Tridemorph	0,002	0,01	(0.01-2)	89	7,0	31,0
243.	Triflumizole	0,002	0,01	(0.01-2)	88	3,6	26,0
244.	Triflumuron	0,002	0,01	(0.01-2)	100	13,0	25,0
245.	Triflusulfuron methyl	0,0025	0,01	(0.01-0.5)	83	4,0	35,5
246.	Triticonazole	0,001	0,01	(0.01-2)	105	8,6	28,9
247.	Tritosulfuron	0,002	0,01	(0.01-2)	89	4,0	25,0
248.	Zoxamide	0,001	0,005	(0.005-2)	95	3,2	15,6
**SUB-METHOD 2**							
1.	2,4,5-T	0,0025	0,01	(0.01 - 0.5)	98	3,93	11
2.	2,4,5-TP (Fenoprop)	0,0025	0,01	(0.01 - 0.5)	96	3,35	9
3.	2,4-D	0,0025	0,01	(0.01 - 0.5)	102	2,90	8
4.	2,4-DB	0,0025	0,01	(0.01 - 0.5)	98	5,42	15
5.	Acibenzolar acid	0,0025	0,01	(0.01 - 0.5)	89	3,19	9
6.	Acifluorfen	0,0025	0,01	(0.01 - 0.5)	88	5,30	15
7.	Aminopyralid	0,0025	0,01	(0.01 - 0.5)	81	4,16	12
8.	Bentazone	0,0025	0,01	(0.01 - 0.5)	95	3,88	11
9.	Bromacil	0,0025	0,01	(0.01 - 0.5)	102	6,70	19
10.	Bromoxynil	0,0025	0,01	(0.01 - 0.5)	94	4,75	13
11.	Clopyralid	0,0025	0,01	(0.01 - 0.5)	85	2,91	8
12.	Dichlorprop	0,0025	0,01	(0.01 - 0.5)	98	3,33	9
13.	Diflufenzopyr	0,0025	0,01	(0.01 - 0.5)	91	5,20	15
14.	Dicamba	0,0025	0,01	(0.01 - 0.5)	93	3,68	10
15.	Diclofop	0,0025	0,01	(0.01 - 0.5)	92	6,78	19
16.	Fenoxaprop-P	0,0025	0,01	(0.01 - 0.5)	93	5,52	16
17.	Fluazifop	0,0025	0,01	(0.01 - 0.5)	98	3,16	9
18.	Fluroxypyr	0,0025	0,01	(0.01 - 0.5)	92	6,17	17
19.	Haloxyfop	0,0025	0,01	(0.01 - 0.5)	95	3,33	9
20.	Imazamox	0,0025	0,01	(0.01 - 0.5)	98	2,20	6

21.	Imazapyr	0,0025	0,01	(0.01 - 0.5)	81	3,30	13
22.	Imazethapyr	0,0025	0,01	(0.01 - 0.5)	92	4,42	13
23.	Ioxynil	0,0025	0,01	(0.01 - 0.5)	96	3,82	11
24.	MCPA	0,0025	0,01	(0.01 - 0.5)	94	4,15	12
25.	MCPB	0,0025	0,01	(0.01 - 0.5)	99	3,43	10
26.	Mecoprop	0,0025	0,01	(0.01 - 0.5)	95	4,19	12
27.	Picloram	0,0025	0,01	(0.01 - 0.5)	88	3,82	11
28.	Triasulfuron	0,0025	0,01	(0.01 - 0.5)	99	2,66	8
29.	Tribenuron Methyl	0,0025	0,01	(0.01 - 0.5)	83	7,63	22
30.	Triclopyr	0,0025	0,01	(0.01 - 0.5)	97	4,98	14
31.	Trinexapac	0,0025	0,01	(0.01 - 0.5)	86	5,19	15
32.	Quinmerac	0,0025	0,01	(0.01 - 0.5)	81	4,70	13
33.	Quizalofop	0,0025	0,01	(0.01 - 0.5)	98	4,17	12

Supplementary Table S4. Multi LC method

No.	Substance name	LOD [mg/kg]	LOQ [mg/kg]	Working Range [mg/kg]	Recoveries [%]	RSD [%]	Measurement Uncertainty [%]
1	Avermectin	0,002	0,01	(0.01-2)	93	14,0	44,0
2	Acephate	0,002	0,01	(0.01-2)	86	7,9	35,8
3	Acetamiprid	0,001	0,005	(0.005-2)	91	3,0	19,1
4	Aclonifen	0,0025	0,01	(0.01-1)	104	8,7	34,9
5	Aldicarb	0,002	0,01	(0.01-2)	97	3,5	11,7
6	Aldicarb sulfone	0,005	0,01	(0.01-2)	105	3,2	19,3
7	Aldicarb sulfoxide	0,001	0,01	(0.01-2)	95	2,5	13,1
8	Ametoctradin	0,001	0,005	(0.005-2)	91	5,5	24,0
9	Amidosulfuron	0,001	0,005	(0.005-2)	80	3,5	41,3
10	Amisulbrom	0,0025	0,01	(0.01-1)	100	6,2	21,8
11	Azadirachtin	0,002	0,01	(0.01-2)	82	13,0	46,0
12	Aziprotryne	0,0025	0,01	(0.01-1)	102	3,8	17,5
13	Azoxystrobin	0,001	0,005	(0.005-2)	91	4,7	27,1
14	BAC C10	0,002	0,01	(0.01-2)	101	5,0	12,0
15	BAC C12	0,002	0,01	(0.01-2)	99	5,0	15,0
16	BAC C14	0,002	0,01	(0.01-2)	82	5,0	41,0
17	BAC C8	0,002	0,01	(0.01-2)	95	3,0	14,0
18	Beflubutamid	0,0025	0,01	(0.01-1)	103	3,7	16,7
19	Bendiocarb	0,0025	0,01	(0.01-1)	103	5,3	19,3

20	Benthiavalicarb isopropyl	0,0025	0,01	(0.01-1)	102	2,5	20,8
21	Benzovindiflupyr	0,001	0,005	(0.005-2)	110	7,0	27,0
22	Bixafen	0,0025	0,01	(0.01-1)	105	4,8	29,3
23	Boscalid	0,002	0,005	(0.005-2)	89	5,2	27,1
24	Bromacil	0,0025	0,01	(0.01-1)	102	2,5	14,4
25	Bromuconazole	0,004	0,01	(0.01-2)	97	7,1	21,0
26	Cadusafos	0,001	0,0025	(0.0025-0.5)	94	3,5	16,2
27	Carbaryl	0,0005	0,005	(0.005-2)	97	4,9	15,4
28	Carbendazim	0,001	0,005	(0.005-2)	94	3,8	16,3
29	Carbetamide	0,0025	0,01	(0.01-1)	101	2,5	15,1
30	Carbofuran	0,0005	0,005	(0.005-2)	98	2,9	14,9
31	Carbofuran 3-hydroxy	0,001	0,005	(0.005-2)	103	6,7	19,8
32	Carbofuran 3-keto	0,001	0,01	(0.01-2)	98	3,0	17,0
33	Carfentrazone-ethyl	0,002	0,01	(0.01-2)	102	10,0	21,0
34	Chlorantraniliprole	0,001	0,005	(0.005-2)	96	5,3	18,7
35	Chloridazon	0,001	0,005	(0.005-2)	97	3,2	15,5
36	Chlorotoluron	0,001	0,005	(0.005-2)	100	3,7	11,1
37	Chlorpyrifos	0,0025	0,01	(0.01-1)	103	3,0	23,3
38	Chlorsulfuron	0,002	0,005	(0.005-2)	76	2,9	48,5
39	Chromafenozide	0,0025	0,01	(0.01-1)	103	2,2	16,2
40	Clodinafop propargyl	0,002	0,01	(0.01-2)	96	3,9	16,0
41	Clofentezine	0,0005	0,005	(0.005-2)	95	3,7	14,1
42	Clothianidin	0,002	0,01	(0.01-2)	103	4,3	16,4
43	Cyantraniliprole	0,002	0,01	(0.01-2)	84	16,0	36,0
44	Cyazofamid	0,001	0,005	(0.005-2)	97	3,3	11,0
45	Cycloxydim	0,001	0,005	(0.005-2)	96	3,2	11,9
46	Cyflufenamid	0,001	0,005	(0.005-2)	93	3,6	17,9
47	Cyflumetofen	0,001	0,005	(0.005-2)	108	7,0	19,0
48	Cymiazol	0,0025	0,01	(0.01-1)	100	2,9	21,6
49	Cymoxanil	0,001	0,005	(0.005-2)	98	3,0	9,2
50	Cyproconazole	0,002	0,01	(0.01-2)	101	3,0	13,3
51	DDAC C8	0,002	0,01	(0.01-2)	102	5,0	15,0
52	DEET	0,002	0,01	(0.01-2)	100	7,9	22,7
53	Demeton-S-methyl	0,001	0,0025	(0.0025-0.5)	93	9,7	17,1

54	Demeton-S-methyl-sulphone	0,001	0,0025	(0.0025-0.5)	94	3,9	15,8
55	Demeton-S-methyl-sulphoxide	0,001	0,0025	(0.0025-0.5)	92	7,0	25,5
56	Desmedipham	0,001	0,01	(0.01-2)	97	3,0	10,6
57	Dicrotophos	0,0025	0,01	(0.01-1)	100	2,6	13,4
58	Diethofencarb	0,0005	0,005	(0.005-2)	101	2,0	8,4
59	Diflubenzuron	0,002	0,005	(0.005-2)	94	7,5	30,2
60	Diflufenican	0,0025	0,01	(0.01-1)	101	3,3	26,0
61	Dimethenamid	0,001	0,005	(0.005-2)	100	8,0	17,0
62	Dimethoate	0,001	0,005	(0.005-2)	97	5,5	17,6
63	Dinoterfuran	0,002	0,01	(0.01-2)	103	6,0	13,0
64	Disulfoton	0,002	0,01	(0.01-2)	82	4,1	47,0
65	Disulfoton sulfon	0,001	0,005	(0.005-2)	96	4,0	14,2
66	Disulfoton sulfoxide	0,001	0,005	(0.005-2)	96	4,0	15,0
67	Diuron	0,0025	0,01	(0.01-1)	102	3,1	12,4
68	DMF	0,0005	0,005	(0.005-2)	100	6,3	18,2
69	DMPF	0,0005	0,005	(0.005-2)	92	2,9	18,9
70	Emamectin	0,0025	0,01	(0.01-1)	103	2,8	22,5
71	Ethametsulfuron-methyl	0,001	0,005	(0.005-2)	96	8,0	20,0
72	Ethiofencarb	0,0025	0,01	(0.01-1)	101	3,0	12,4
73	Ethirimol	0,0025	0,01	(0.01-1)	94	2,9	18,6
74	Etoxazole	0,0005	0,005	(0.005-2)	88	3,6	25,4
75	Famoxadone	0,0025	0,01	(0.01-1)	100	4,1	30,9
76	Fenamidone	0,001	0,005	(0.005-2)	94	3,2	15,6
77	Fenamiphos	0,001	0,005	(0.005-2)	94	3,3	15,9
78	Fenamiphos sulfoxide	0,0005	0,005	(0.005-2)	96	2,0	10,6
79	Fenamiphos sulphone	0,001	0,005	(0.005-2)	99	3,0	10,4
80	Fenbuconazole	0,002	0,005	(0.005-2)	102	10,3	33,7
81	Fenfuram	0,0025	0,01	(0.01-1)	104	3,0	16,5
82	Fenhexamid	0,0025	0,01	(0.01-1)	94	5,3	18,9
83	Fenobucarb	0,0025	0,01	(0.01-1)	102	4,1	15,1
84	Fenoxaprop-P-ethyl	0,0005	0,005	(0.005-2)	96	2,6	12,8
85	Fenpropidin	0,0025	0,01	(0.01-1)	102	2,7	16,5

86	Fenpropimorph	0,001	0,005	(0.005-2)	98	3,8	12,0
87	Fenpyroximate	0,0005	0,005	(0.005-2)	99	3,7	15,7
88	Fensulfothion	0,001	0,0025	(0.0025-0.5)	92	4,7	20,3
89	Fensulfothion oxon	0,001	0,0025	(0.0025-0.5)	92	4	19,6
90	Fensulfothion oxon sulphone	0,001	0,0025	(0.0025-0.5)	99	5,7	16,4
91	Fensulfothion sulphone	0,001	0,0025	(0.0025-0.5)	94	4,8	18,7
92	Fenthion	0,0025	0,01	(0.01-1)	103	9,7	39,5
93	Fenthion oxon	0,002	0,01	(0.01-2)	92	2,8	17
94	Fenthion oxon sulphone	0,002	0,01	(0.01-2)	88	2,4	25
95	Fenthion sulfoxide	0,0025	0,01	(0.01-1)	105	3,2	14,3
96	Fenthion sulphone	0,002	0,01	(0.01-2)	91	3,5	20,0
97	Flazasulfuron	0,001	0,005	(0.005-2)	96	8,0	20,0
98	Flonicamid	0,001	0,005	(0.005-2)	94	2,7	15,1
99	Florasulam	0,002	0,01	(0.01-2)	84	5,0	43,0
100	Flufenacet	0,001	0,005	(0.005-2)	99	3,4	10,6
101	Flufenoxuron	0,001	0,005	(0.005-2)	92	5,9	29,6
102	Fluopicolide	0,001	0,005	(0.005-2)	99	8,0	16,0
103	Fluopyram	0,001	0,005	(0.005-2)	101	4,0	11,0
104	Fluoxastrobin	0,001	0,005	(0.005-2)	100	7,0	22,5
105	Flupyradifurone	0,002	0,01	(0.01-2)	81	19,0	39,0
106	Flurochloridone	0,005	0,01	(0.01-2)	96	9,2	27,6
107	Flutianil	0,002	0,01	(0.01-2)	90	4,6	24,0
108	Flutolanil	0,001	0,005	(0.005-2)	98	2,0	6,6
109	Flutriafol	0,001	0,01	(0.01-2)	99	3,0	9,4
110	Fluxapyroxad	0,0025	0,01	(0.01-1)	102	5,5	19,3
111	Foramsulfuron	0,001	0,005	(0.005-2)	82	12,8	46,0
112	Formetanate	0,001	0,01	(0.01-2)	101	4,2	16,9
113	Fosthiazate	0,0025	0,01	(0.01-1)	102	2,9	10,0
114	Fuberidazole	0,001	0,005	(0.005-2)	95	2,7	15,0
115	Halofenozide	0,002	0,01	(0.01-2)	96	2,4	20,0
116	Hexaflumuron	0,001	0,005	(0.005-2)	102	9,0	21,0
117	Hexytiazox	0,001	0,005	(0.005-2)	97	3,8	12,5
118	Imazalil	0,001	0,01	(0.01-2)	98	1,7	8,4
119	Imazapic	0,002	0,01	(0.01-2)	101	5,0	16,0
120	Imidacloprid	0,001	0,01	(0.01-2)	99	3,2	10,0

121	Indoxacarb	0,001	0,005	(0.005-2)	100	5,4	16,3
122	Iodosulfuron methyl	0,0025	0,01	(0.01-1)	84	4,5	34,6
123	Ipconazole	0,0025	0,01	(0.01-1)	98	5,4	21,0
124	Iprovalicarb	0,001	0,005	(0.005-2)	94	3,4	16,0
125	Isofetamid	0,002	0,01	(0.01-2)	100	2,3	17,0
126	Isoprocarb	0,0025	0,01	(0.01-1)	103	3,0	20,0
127	Isoprothiolane	0,0025	0,01	(0.01-1)	101	9,3	18,0
128	Isoproturon	0,001	0,005	(0.005-2)	99	2,5	10,2
129	Isopyrazam	0,001	0,005	(0.005-2)	98	1,7	13,9
130	Isoxaben	0,001	0,005	(0.005-2)	110	5,0	26,0
131	Isoxaflutole	0,001	0,005	(0.005-2)	97	14,0	29,0
132	Isoxathion	0,001	0,005	(0.005-2)	110	6,0	26,0
133	Lenacil	0,001	0,01	(0.01-2)	93	5,2	20,4
134	Linuron	0,001	0,005	(0.005-2)	98	8,0	24,4
135	Lufenuron	0,002	0,01	(0.01-2)	86	11,0	44,0
136	Malaoxon DITIOCARBAMATES	0,001	0,005	(0.005-2)	96	2,1	15,1
137	Malathion	0,001	0,01	(0.01-2)	102	4,2	13,6
138	Mandipropamid	0,001	0,005	(0.005-2)	93	2,7	16,1
139	Metaflumizone	0,002	0,01	(0.01-2)	100	9,0	26,0
140	Metalaxyl	0,0005	0,005	(0.005-2)	98	2,8	10,8
141	Metamitron	0,002	0,01	(0.01-2)	94	2,7	13,7
142	Metazachlor	0,002	0,01	(0.01-2)	94	2,3	16,0
143	Methamidophos	0,002	0,01	(0.01-2)	93	2,6	15,7
144	Methiocarb	0,0005	0,005	(0.005-2)	102	3,4	10,9
145	Methiocarb sulphone	0,001	0,01	(0.01-2)	92	6,3	23,8
146	Methiocarb sulphoxide	0,0005	0,005	(0.005-2)	93	1,8	15,4
147	Methomyl	0,001	0,01	(0.01-2)	99	3,6	10,6
148	Methoprotryne	0,002	0,01	(0.01-2)	112	5,0	26,0
149	Methoxyfenozide	0,002	0,005	(0.005-2)	100	2,2	9,3
150	Metobromuron	0,002	0,01	(0.01-2)	100	5,0	13,0
151	Metolachlor-S	0,001	0,005	(0.005-2)	93	4,9	19,5
152	Metosulam	0,0025	0,01	(0.01-2)	92	13,7	44
153	Metoxuron	0,0025	0,01	(0.01-1)	101	2,4	11,4
154	Metrafenone	0,001	0,005	(0.005-2)	99	3,2	10,1

155	Metsulfuron-methyl	0,002	0,005	(0.005-2)	79	4,9	44,9
156	Monocrotophos	0,001	0,005	(0.005-2)	100	3,7	13,3
157	Monuron	0,0025	0,01	(0.01-1)	105	4,9	18,1
158	Napropamide	0,001	0,005	(0.005-2)	98	4,1	13,8
159	Nicosulfuron	0,001	0,005	(0.005-2)	88	11,0	32,0
160	Nicotine	0,0025	0,01	(0.01-2)	93	8,2	26,9
161	Nitenpyram	0,002	0,01	(0.01-2)	106	8,0	18,0
162	Novaluron	0,001	0,005	(0.005-2)	99	14,0	35,0
163	Omethoate	0,001	0,0025	(0.0025-2)	90	7,4	28,6
164	Oxadixyl	0,001	0,005	(0.005-2)	97	3,2	12,5
165	Oxamyl	0,001	0,005	(0.005-2)	93	1,8	14,6
166	Oxycarboxin	0,0025	0,01	(0.01-1)	100	2,8	12,6
167	Paraoxon-methyl	0,001	0,005	(0.005-2)	101	3,5	10,2
168	Parathion	0,001	0,01	(0.01-2)	100	8,6	27,5
169	Parathion-methyl	0,0025	0,01	(0.01-1)	100	11,7	33,7
170	Pencycuron	0,001	0,005	(0.005-2)	96	5,7	19,7
171	Pendimethalin	0,002	0,005	(0.005-2)	102	2,0	13,2
172	Penflufen	0,002	0,01	(0.01-2)	117	6,0	35,0
173	Penthiopyrad	0,0025	0,01	(0.01-1)	103	3,7	14,3
174	Pethoxamid	0,0025	0,01	(0.01-0.5)	94	6,1	20,5
175	Phenmedipham	0,001	0,01	(0.01-2)	97	4,4	14,1
176	Phenthoate	0,001	0,005	(0.005-2)	96	3,0	12,0
177	Phosmet	0,001	0,005	(0.005-2)	97	2,8	10,0
178	Phosmet okson	0,002	0,01	(0.01-2)	109	7,7	27,6
179	Phoxim	0,002	0,01	(0.01-2)	104	8,0	22,0
180	Pinoxaden	0,0005	0,005	(0.005-2)	92	3,7	19,6
181	Piperonyl butoxide	0,0025	0,01	(0.01-1)	103	2,5	16,3
182	Prochloraz	0,001	0,005	(0.005-2)	98	2,7	8,5
183	Prochloraz BTS 44595	0,0025	0,01	(0.01-1)	104	3,0	21,6
184	Prochloraz BTS 44596	0,0025	0,01	(0.01-1)	101	3,4	14,8
185	Propamocarb	0,001	0,005	(0.005-2)	80	3,8	42,1
186	Propaquizafop	0,001	0,005	(0.005-2)	98	3,1	11,5
187	Propoxur	0,0025	0,01	(0.01-1)	101	2,6	14,9
188	Propoxycarbazone	0,0025	0,01	(0.01-1)	85	4,6	33,0
189	Proquinazid	0,001	0,005	(0.005-2)	97	1,3	10,2

190	Prosulfocarb	0,001	0,005	(0.005-2)	96	2,8	15,7
191	Prosulfuron	0,001	0,005	(0.005-2)	94	19,0	36,0
192	Pymetrozine	0,005	0,02	(0.02-2)	79	2,8	49
193	Pyridaben	0,001	0,005	(0.005-2)	93	2,7	15,8
194	Pyridafol	0,002	0,01	(0.01-2)	79	3,9	47
195	Pyrifenox	0,002	0,01	(0.01-2)	92	8,0	27,0
196	Pyriproxyfen	0,0025	0,01	(0.01-1)	104	2,7	16,7
197	Pyroquilon	0,0025	0,01	(0.01-1)	101	2,3	11,5
198	Pyroxsulam	0,001	0,005	(0.005-2)	94	7,0	18,0
199	Quinclorac	0,002	0,01	(0.01-2)	114	17,0	38,0
200	Quinoclanmine	0,0025	0,01	(0.01-1)	105	5,2	26,8
201	Quizalofop-ethyl	0,001	0,005	(0.005-2)	96	2,2	11,8
202	Rimsulfuron	0,001	0,01	(0.01-2)	84	4,2	44,7
203	Rotenone	0,0025	0,01	(0.01-1)	103	3,5	27,0
204	Saflufenacil	0,001	0,005	(0.005-2)	102	12,0	26,0
205	Silthiofam	0,001	0,005	(0.005-2)	95	6,2	20,4
206	Spinetoram	0,0025	0,01	(0.01-1)	102	2,6	22,0
207	Spinosyn A	0,001	0,005	(0.005-2)	94	2,5	13,5
208	Spinosyn D	0,001	0,005	(0.005-2)	95	1,9	13,7
209	Spirodiclofen	0,001	0,005	(0.005-2)	92	2,5	17,7
210	Spirotetramat	0,001	0,005	(0.005-2)	95	3,5	15,8
211	Spirotetramat enol	0,001	0,005	(0.005-2)	78	3,9	48,1
212	Spirotetramat enol-glucoside	0,002	0,005	(0.005-2)	85	3,9	42,0
213	Spirotetramat ketohydroxy	0,001	0,005	(0.005-2)	92	9,4	18,7
214	Spirotetramat monohydroxy	0,001	0,005	(0.005-2)	94	5,4	19,4
215	Spiroxamine	0,001	0,005	(0.005-2)	94	2,7	14,4
216	Sulfometuron methyl	0,002	0,005	(0.005-2)	77	3,5	47,4
217	Sulfosulfuron	0,0025	0,01	(0.01-1)	90	4,4	25,8
218	Sulfoxaflor	0,002	0,01	(0.01-2)	97	12,0	10,0
219	Tebuconazole	0,002	0,01	(0.01-2)	97	7,3	24,4
220	Tebufenozide	0,001	0,005	(0.005-2)	93	2,5	17,1
221	Tebufenpyrad	0,001	0,005	(0.005-2)	99	5,4	17,0
222	Teflubenzuron	0,005	0,01	(0.01-2)	93	6,7	23,7
223	Tepraloxydim	0,005	0,01	(0.01-2)	97	6,5	19,6

224	Terbufos	0,0025	0,01	(0.01-0.5)	94	6,1	21,0
225	Terbufos oxon	0,002	0,01	(0.01-2)	85	3,0	32,0
226	Terbufos sulphone	0,0025	0,01	(0.01-0.5)	93	5,9	21,9
227	Terbufos sulphoxide	0,001	0,005	(0.005-2)	93	7,8	26,7
228	Terbuthylazine	0,001	0,005	(0.005-2)	97	2,2	9,4
229	Thiabendazole	0,001	0,005	(0.005-2)	93	1,7	15,5
230	Thiacloprid	0,001	0,005	(0.005-2)	98	2,0	9,7
231	Thiamethoxam	0,001	0,005	(0.005-2)	97	2,2	12,5
232	Thiencarbazone-methyl	0,001	0,005	(0.005-2)	97	14,0	33,0
233	Thifensulfuron-methyl	0,002	0,01	(0.01-2)	76	4,0	49,0
234	Thiodicarb	0,0005	0,005	(0.005-2)	93	2,7	15,7
235	Thiometon	0,0025	0,01	(0.01-1)	96	7,2	22,0
236	Thiophanate-methyl	0,001	0,005	(0.005-2)	97	1,9	10,6
237	Tolfenpyrad	0,002	0,01	(0.01-2)	80	2,0	39,0
238	Topramezone	0,002	0,01	(0.01-2)	98	5,0	11,0
239	Tralkoxydim	0,0025	0,01	(0.01-1)	92	3,3	22,0
240	Trichlorfon	0,002	0,01	(0.01-2)	96	9,0	24,0
241	Tricyclazole	0,0025	0,01	(0.01-1)	97	2,4	15,8
242	Tridemorph	0,002	0,01	(0.01-2)	89	7,0	31,0
243	Triflumizole	0,002	0,01	(0.01-2)	88	3,6	26,0
244	Triflumuron	0,002	0,01	(0.01-2)	100	13,0	25,0
245	Triflusulfuron methyl	0,0025	0,01	(0.01-0.5)	83	4,0	35,5
246	Triticonazole	0,001	0,01	(0.01-2)	105	8,6	28,9
247	Tritosulfuron	0,002	0,01	(0.01-2)	89	4,0	25,0
248	Zoxamide	0,001	0,005	(0.005-2)	95	3,2	15,6
